# New stiletto flies from New Caledonia (Therevidae, Agapophytinae)

**DOI:** 10.3897/zookeys.984.53587

**Published:** 2020-11-04

**Authors:** Michael E. Irwin, Shaun L. Winterton, Mark A. Metz

**Affiliations:** 1 Emeritus, University of Illinois, Urbana-Champaign, Illinois, USA University of Illinois Urbana-Champaign United States of America; 2 University of Arizona, Tucson, USA University of Arizona Tucson United States of America; 3 California State Collection of Arthropods, California Department of Food & Agriculture, Sacramento, California, USA California State Collection of Arthropods Sacramento United States of America; 4 USDA, ARS, Systematic Entomology Laboratory, Beltsville, Maryland, USA Systematic Entomology Laboratory Beltsville United States of America

**Keywords:** Asiloidea, Australasia, Diptera, Oceania

## Abstract

Stiletto-flies (Diptera: Therevidae) are highly diverse and species-rich in Australia and New Zealand, yet relatively few species have been recorded from neighbouring Papua New Guinea, Indonesia and throughout the remainder of Oceania. Indeed, in New Caledonia only a single species of the widely distributed Australasian genus *Anabarhynchus* Macquart (Therevinae) is previously known. Herein we describe two new agapophytine genera (i.e., *Jeanchazeauia***gen. nov.**, *Calophytus***gen. nov.**), together comprising nine charismatic new species; this represents a first record of the subfamily from New Caledonia. The new genera and species are described and figured.

## Introduction

Stiletto flies (Diptera: Therevidae) comprise more than 1200 species in ca. 130 genera worldwide, with the family divided into four subfamilies: Therevinae, Xestomyzinae, Phycusinae, and Agapophytinae (Winterton et al. 2016). They are present in all major biogeographical regions and are particularly common and diverse in mesic to xeric habitats. The greatest species-level diversity is in the subfamily Therevinae, followed by Agapophytinae, with the other two smaller subfamilies much less species-rich. Individuals of Agapophytinae can be distinguished from other therevids by the following characteristics: 1) absence of adpressed scale-like setae on the femora; 2) female genitalia internally with three spermathecae and a spermathecal sac; and 3) male genitalia always have an articulated inner gonocoxal process and an aedeagus with a strongly forked ventral apodeme (Winterton et al. 2001, 2016). The presence of adpressed scale-like setae on the femora is a unique characteristic found only in Therevinae and is the main external feature used to differentiate Therevinae from other subfamilies; all other important characters used to separate subfamilies are internal and genitalic. Caution should be used though in the Australasian and Neotropical regions as one group of therevine genera (the *Anabarhynchus* genus-group: *Anabarhynchus* Macquart, 1848; *Megathereva*, Lyneborg, 2001; *Microthereva* Malloch, 1932; *Peralia* Malloch, 1932) typically lack adpressed setae on the femora, although they exhibit all other therevine characteristics.

While Therevinae are cosmopolitan in their distribution, Agapophytinae are a distinctively southern hemisphere radiation found in Australia, Indonesia, New Zealand, Papua New Guinea and South America. The bulk of the agapophytine generic diversity occurs in Australasia, although four genera have been described from South America (Webb et al. 2013; Irwin and Winterton 2020). Relatively few species are known from Papua New Guinea and Indonesia. Here we describe two new genera of Agapophytinae from near-by New Caledonia, an archipelago in the South Pacific comprised of Grande Terre, the Loyalty Islands, and a series of smaller islands. It is located in the Coral Sea southeast of Vanuatu, 1200 km to the east of Australia and 2400 km north of New Zealand. One subspecies of the aforementioned genus *Anabarhynchus* (i.e., *A.hyalipennisvaricincta* (Bigot, 1860)) is distributed widely in New Caledonia and Vanuatu; the other subspecies (*A.hyalipennishyalipennis* (Macquart, 1846)) is distributed widely throughout Australia, including Lord Howe and Norfolk Islands (Lyneborg 2001). The remaining stiletto fly fauna newly described here comprises two new genera containing nine new species endemic to New Caledonia. *Calophytus* gen. nov. and *Jeanchazeauia* gen. nov. were previously included in a phylogenetic study of Therevidae by Winterton et al. (2016) (as undescribed genus ‘NC’), who recovered them as members of the *Taenogera* genus-group (Agapophytinae) (Fig. 4). The New Caledonia genera were recovered as sister to the remaining *Taenogera* genus-group, which includes the genera *Collessiama* Lambkin, 2013, *Ectinorhynchus* Macquart, 1850, *Evansomyia* Mann, 1928, *Taenogera* Kröber, 1912 and *Taenogerella* Winterton & Irwin, 1999b (see Winterton et al. 1999b; Winterton et al. 2016). It also includes the genus *Squamopygia* Kröber, 1928, and while this genus was not included in their molecular phylogenetic study by Winterton et al. (2016) it was included in the morphological phylogeny by Winterton et al. (1999b), where it was placed as sister to *Ectinorhynchus* and *Evansomyia*. Winterton et al. (2016) hypothesised that the common ancestor of the New Caledonia genera and the Australasian genera of the *Taenogera* genus-group diverged during the end of the Palaeogene or the start of the Neogene (12–23 MYA). This suggests that this New Caledonia stiletto-fly fauna is a relative recent radiation of closely related taxa whose common ancestor arrived via dispersal from Papua New Guinea, New Zealand or Australia.

## Materials and methods

Adult morphological terminology follows Cumming and Wood (2017) with additional therevoid-specific genitalic morphology according Winterton et al. (1999a, b, c) and Winterton (2006). Genitalia were macerated in 10% KOH or lactic acid to remove soft tissue, then rinsed in dilute glacial acetic acid or distilled water, respectively, and dissected in 80% ethanol or glycerine. Genitalia preparations were placed in glycerine in a genitalia vial mounted on the pin beneath the specimen. Specimen images were taken at different focal points using a digital camera and subsequently combined into a serial montage image. Specimens have a unique number attached to them usually on a separate yellow label with ME Irwin Therevidae Specimen Number “MEI99999”. These are contained in a specimen database ‘Mandala’ (Kampmeier and Irwin 2009). All new nomenclatural acts are registered in ZooBank (Pyle and Michel 2008). Specimens are deposited in the following institutions:

**BPBM**Bishop Museum, Honolulu, Hawai’i, USA;

**CSCA** California Food and Agricultural Department, Sacramento, California, USA;

**CNC** Canadian National Collection of Insects, Arachnids & Nematodes, Agriculture & Agri-Food Canada, Ottawa, Ontario, Canada;

**FSCA**Florida State Collection of Arthropods, Gainesville, Florida, USA;

**MNHN** Muséum national d’Histoire naturelle, Paris, Île-de-France, France;

**NHRS**Naturhistoriska Riksmuseet, Stockholm, Uppland, Sweden;

**QM**Queensland Museum, Brisbane, Queensland, Australia;

**UMSP**University of Minnesota, Saint Paul, Minnesota, USA.

## Taxonomy

A key to genera of stiletto flies of New Caledonia is presented. While *Anabarhynchushyalipennisvaricincta* is a widely distributed species in both New Caledonia and Vanuatu, *Calophytus* gen. nov. and *Jeanchazeauia* gen. nov. are entirely endemic to Grande Terre. No other Therevidae have been recorded from New Caledonia. A revised key to Australasian genera is needed but is beyond the scope of this study.

### Key to genera of New Caledonian Therevidae

**Table:** 

1	Antennae much shorter than head length, flagellum strongly conical; wing markings largely absent; 2 pairs of scutellar macrosetae; fore- and midfemora with anteroventral and posteroventral macrosetae present; female sternite VIII with subapical, forked process projecting ventrally; two spermathecae present	***Anabarhynchus* (i.e., *A.hyalipennisvaricincta*)**
–	Antennae slightly to much longer than head, flagellum elongate and tapered apically; wing markings distinct, derived from infuscation of wing membrane and dark microtrichia; single pair of scutellar macrosetae; fore- and midfemora without macrosetae; female sternite VIII without projecting process; three spermathecae present	**2**
2	Anepisternum glabrous, lacking silver pubescence (e.g., Fig. 9); wing with basal radial cell (br) bisected along its length by a stripe of microtrichia along wing fold (Fig. 8); scutellum polished or with sparse pubescence; hind femur lacking subapical anteroventral setae; single posteroventral macroseta midway along hind femur; female tergite VIII quadrangular-shaped (Fig. 23A)	***Calophytus* gen. nov.** (6 spp.) (Figs 9, 11, 13, 15, 16, 19)
–	Anepisternum with silver pubescence (e.g., Fig. 28); wing with cell *br* not bisected by stripe of microtrichia, when microtrichia present then irregular and not arranged as a single stripe along wing fold (Fig. 7G, H); scutellum often with dense, matte pubescence; hind femur with one or more distinct subapical anteroventral macrosetae; posteroventral macrosetae present as series along hind femur but very small and sometimes barely evident; female tergite VIII short, ‘T’-shaped with anteromedial projection elongate, often with robust, up-curved setae laterally (Fig. 23B–D)	***Jeanchazeauia* gen. nov.** (3 spp.) (Figs 24, 25, 28, 29)

#### Calophytus gen. nov.

Taxon classificationAnimaliaTherevidaeAgapophytinae

B662223F-2079-50ED-B60E-F236FB0F33BD

http://zoobank.org/CB02979A-20AF-4C4E-BFB1-B8D05668523C

##### Type species.

*Calophytuschazeaui* sp. nov., here designated.

##### Diagnosis.

Antennae elongate, longer than head, narrow cylindrical; frons wider than ocellar tubercle at narrowest point with only slight sexual dimorphism; dorsocentral macrosetae absent; velutum patches absent on femora; single posteroventral macroseta present midway along hind femur; wing with hyaline areas often free of microtrichia; distinct stripe of dark microtrichia bisecting cell br along wing fold; male genitalia with gonocoxites without velutum patch and medial atrium (gonocoxites proximal medially).

##### Description.

Antenna longer than head; flagellum cylindrical, slightly tapered distally, shorter than combined scape and pedicel length, rarely longer (in *C.webbi* sp. nov.); scape cylindrical, usually elongate; head shape in profile with length and height subequal; frons glossy, slightly protruding anterior to eye around base of antennae; frons wider than ocellar tubercle at narrowest point, only slight sexual dimorphism in frons width with space between eyes slightly narrower in male; parafacial without setae, postocular macrosetae in both sexes arranged in a single row dorsally, rarely scattered dorsomedially on occiput (in *C.grandiosus* sp. nov.); prosternum without setae; fore and hind femoral velutum patches absent; femoral macrosetae absent except for single posteroventral macroseta present midway along hind femur; posterior surface of mid coxa without setae; hind femur and tibia relatively longer than that of fore and mid legs; post-spiracular setae absent; scutal chaetotaxy (pairs): notopleural (np), 1–2; supra alar (sa), 1; post alar (pa), 1; dorsocentral (dc), 0; scutellar (sc), 1; wing cell m_3_ open to margin; wing vein R_2+3_ smoothly curved or straight to wing margin, wing with markings faint to strongly banded, hyaline areas often free of microtrichia; distinct stripe of dark microtrichia bisecting cell br along wing fold; abdominal tergite II with all setae uniform and regular in length. Male genitalia with dorsal apodeme of aedeagus ‘T’-shaped, distiphallus narrow, straight, ventral apodeme forked; gonocoxite with velutum patch absent, posteromedial margins proximal to each other; inner gonocoxal process (igp) present and articulated; ventral lobe small, rounded apically. Female genitalia with tergite VIII elongate, quadrangular, anteromedial process narrow; tergite VII lacking anteromedial process; acanthophorite setae as two sets (A1 & A2), A1 enlarged and rounded apically, A2 series prominent; sternite VIII emarginate posteromedially, flattened; spermathecal sac present, not lobed; three sac-like spermathecae present, joined to spermathecal sac duct near junction with bursa copulatrix.

##### Etymology.

Derived from the Greek *kallos*, beauty; and *phyton*, plant, and relating to the charismatic appearance of the flies and the natural areas where they are collected in New Caledonia. The name also associates this genus with New Caledonia via the Proto-Celtic root of *kal*, hard, pertaining to the Roman name for the original provenance of Caledones in Britannia. Gender is masculine.

##### Comments.

All species of *Calophytus* gen. nov. are medium to large stiletto flies, with heads mostly glossy with striking patches of silver pubescence on the parafacial and pleuron, banded wings and antennae longer than the head. Distinctive to members of *Calophytus* gen. nov. is the presence of a single posteroventral macroseta midway along the hind femur and a stripe of microtrichia along the wing fold bisecting an otherwise glabrous wing cell br. These two characteristics are also found in the genus *Squamopygia* from Australia and Papua New Guinea and appear unique to these two genera amongst all Therevidae; they are notably absent in *Jeanchazeauia* gen. nov. While Winterton et al. (2016) recovered a clear sister-group relationship between *Calophytus* gen. nov. and *Jeanchazeauia* gen. nov. (included in their study as undescribed genus ‘NC’) (Fig. 4), they did not include the rarely collected *Squamopygia*. The two aforementioned characters found only in *Squamopygia* and *Calophytus* gen. nov. also support a likely sister-group relationship between these two genera. *Squamopygia* is a monotypic genus with a single species from Northern Australia; two undescribed species are known from southern Australia and Papua New Guinea. *Calophytus* gen. nov. can be separated from *Squamopygia* by the absence of subapical anteroventral macrosetae on the hind femur (present in *Squamopygia*), absence of dorsocentral macrosetae (present in *Squamopygia*) and by the width of the male frons. The sexual dimorphism in male frons width is more pronounced in *Squamopygia* with the eyes being contiguous. In *Calophytus* gen. nov. (and *Jeanchazeauia* gen. nov.) the male frons is only slightly narrower than that of the female, with the frons wider than the ocellar tubercle in both sexes. *Squamopygia* also has banded wings similar to *Calophytus* gen. nov., but the hyaline areas of the wing membrane retain extensive pale microtrichia. The male and female genitalia of *Squamopygia* and *Calophytus* are very similar. *Calophytusgrandiosus* sp. nov. is rather distinct from all other *Calophytus* species, differing in the arrangement of postocular macrosetae, wing banding pattern and body size, supporting its position as sister to the rest of the genus (Fig. 4). *Calophytus* species have been collected in all of the main habitat types throughout New Caledonia, including rainforest, maquis scrub and dry sclerophyll forest (Figs 22, 31, 32).

##### Included species.

*Calophytuschazeaui* sp. nov., *C.grandiosus* sp. nov., *C.matilei* sp. nov., *C.monteithi* sp. nov., *C.schlingeri* sp. nov., *C.webbi* sp. nov.

### Key to species of *Calophytus* gen. nov.

**Table:** 

1	Postocular macrosetae densely scattered over entire occiput; maxillary palpi large, dark brown, bulbous; labellum brown; large, showy, banded-winged species, infuscation well demarcated (Figs 2, 11)	***C.grandiosus* sp. nov.**
–	Postocular macrosetae concentrated as a single row along postocular ridge; maxillary palpi small, thin with base yellow and apical third light to dark brown; labellum yellowish apically; smaller, yellow to brown species, wing markings typically irregular or fenestrate, sometimes with diffuse margins, not distinctly banded	**2**
2	Scutum predominantly black, sometimes orange laterally along notopleuron (e.g., Fig. 6C); scutellum black; abdomen ground colour dark brown, male abdomen with extensive silver velutum pubescence (e.g., Fig. 16B)	**3**
–	Scutum predominantly dark yellow orange, with dark dorsocentral stripes merging posteriorly (Fig. 6D); scutellum dark yellow, sometimes with brown suffusion (tint) medially; abdomen predominantly dark yellow, male (where known) abdomen lacking silver pubescence (Fig. 9B)	**4**
3	Parafacial with silvery pubescence as an acute wedge-shaped patch along eye margin below antennal insertion, replaced by matte black pubescence ventrally along margin of buccal area; mid- and hind coxae extensively orange, midcoxa almost completely orange (Fig. 13)	***C.matilei* sp. nov.**
–	Parafacial with silvery pubescence as an elongate wedge-shaped patch along eye margin below antennal insertion and extending ventrally along margin of buccal area; mid- and hind coxae extensively dark brown to black, often only apices orange (Fig. 16)	***C.schlingeri* sp. nov.**
4	Antennal flagellum longer than scape (Figs 5E, 20); costal cell of wing only with slight yellowish infuscation (Fig. 7E); scutellum with sparse silver pubescence; relatively small, diminutive individuals (body length: ca. 5.5–8.0 mm) (Fig. 19)	***C.webbi* sp. nov.**
–	Antennal flagellum shorter than scape (Fig. 5A–D); costal cell of wing with distinct yellow infuscation (Fig. 7A, F); scutellum lacking silver pubescence; relatively larger individuals (body length: ca. 7.0–10.0 mm)	**5**
5	Postocular macrosetae orange to brown; pleuron orange; frons around base of antenna, face, and parafacial yellow orange (Fig. 15)	***C.monteithi* sp. nov.**
–	Postocular macrosetae black; dorsal half of pleuron dark brown, orange in ventral half; frons, face and parafacial brown to black (Fig. 9)	***C.chazeaui* sp. nov.**

#### Calophytus chazeaui sp. nov.

Taxon classificationAnimaliaTherevidaeAgapophytinae

27CC9F6B-23D6-5754-AF07-87915FEAA0FB

http://zoobank.org/F8FF4B02-80A9-4E0C-A5AA-8D52A274221A

Figs 1 , 5A , 7A , 8A , 9 , 10 , 22 , 31A

##### Diagnosis.

Scutum with large areas of orange; wing with dark infuscation over discal cell and parts of adjoining cells, as well most of apical third of wing; costal cell with yellow infuscation; male abdomen without silver velutum pubescence; occipital macrosetae black, arranged in single row dorsally in both sexes; legs yellow; flagellum shorter than scape.

##### Description.

Length 7.8–10.6 mm. *Head*. Glossy black, smooth. Frons smooth, raised around base of antennae. Eyes separated at narrowest point by 3× width of median ocellus in both sexes. Parafacial with silver pubescence laterally along eye margin. Occiput silver pubescent laterally and ventrally onto gena, with fine white setae ventrally. Postocular macrosetae black, arranged in a single row dorsally. Scape equal to head length; mostly brown-black, dark yellow basally with numerous short, brown filiform setae along entire length. Basal flagellomere ½× length of scape; with short, black, filiform setae on basal 1/2. Second flagellomere apical, cylindrical, 1/7× length of basal flagellomere. Third flagellomere ½× length of second. Style subequal in length to third flagellomere, spiculate. Palpus widened subapically; apex slightly acuminate; yellow basally, black at apex; yellow pubescent basally, black pubescent at apex, black setose at apex. Mouthparts yellowish; brown setose. *Thorax*. Dark yellow to orange, except scutum with dark brown dorsocentral stripes, narrow anteriorly, widening and converging posteriorly; dorsal pleuron dark brown; posterodorsal katepisternum, metanepisternum, metepimeron, meron and metakatepisternum silver pubescent; scutellum brown; macrosetae black (np: 2, sa: 1, pa: 1, dc: 0, sc: 1); scutum with short black setae; katatergite, lateral postpronotum, cervical sclerite, proepisternum, and lateral prosternum with pale setae. *Legs*. Yellow except with small black spot ventrally at union of femur and trochanter, tarsi brownish; coxae with pale filiform setae anteriorly. *Wing*. Membrane mostly hyaline; costal cell hyaline with pale yellow infuscation; subcostal and radial cells adjacent to pterostigma and entire wing apex with light brown infuscation with dark microtrichia; membrane with extensive areas bare of microtrichia; pterostigma brown. Venation brown, cell m_3_ widely open at wing margin. Haltere completely yellow. *Abdomen*. Dark yellow and brown, proportions highly variable. Tergites II and III yellow with brown medial stripe, brown laterally; tergites IV–VII dark brown; sternites I–VI yellow, sternite VII brown; sparse short, black, fine setae on all segments, longer laterally. *Genitalia*. Male: tergite VIII emarginated posteriorly; setose laterally. Sternite VIII ovate; posterior margin setose. Epandrium with posterolateral corners extending posteriorly level with end of cerci; setae uniformly short and dark. Cercus subtriangular, rounded apically. Subepandrial sclerite narrow, half width of epandrium; partially sclerotised, lateral margins more strongly so. Gonocoxites elongate, rounded with large subtriangular outer gonocoxal process, dark setae erect and sparse, longer ventrally; inner gonocoxal process very narrow, curved medially; gonostylus robust, spatulate, forked as pair of tooth-like lobes apically. Aedeagus with distiphallus relatively straight, fine spines apically; dorsal apodeme forked, ‘T’-shaped; ventral apodeme forked, arms narrow and divergent; lateral ejaculatory apodemes narrow and band-like around anterior of basiphallus; ejaculatory apodeme relatively small, roughly cylindrical, basiphallus subcordate. Female: acanthophorite spines light brown. Sternite X triangular, truncate posteriorly. Furca rounded anteriorly, elongated and acuminate posteriorly. Spermathecal duct equal to four furcal lengths. Spermathecal sac duct two furcal lengths; spermathecal sac ovoid, longer than wide; 2.0× length of spermathecal sac duct. *Variation*. Some individuals with apical 2/3 of scape brown; notum dark with dorsocentral stripes evident; amount of dark brown colour on pleuron varying with most of pleuron dark; wing infuscation variable from slightly tinged to intensely dark; abdominal colouration variable, tergites III–VII from completely brown to lighter; sternite VII brown on posterior margin.

**Figure 1.**
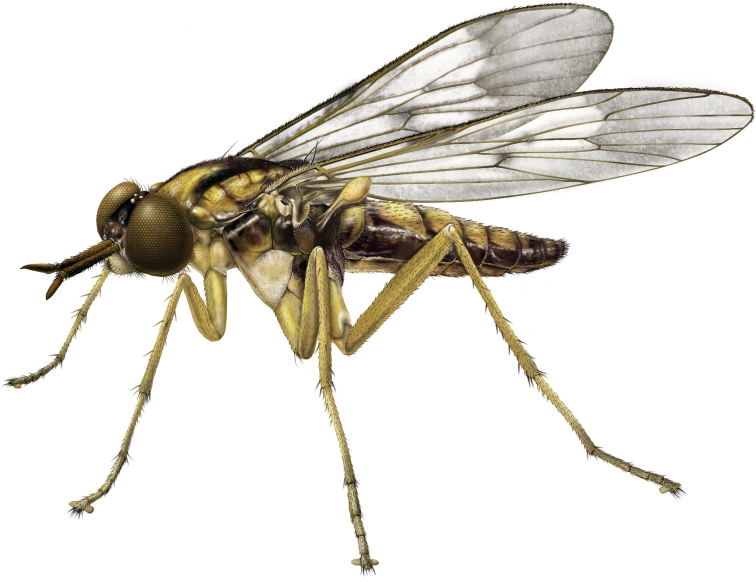
*Calophytuschazeaui* sp. nov., male habitus (specimen: MEI123497) (artistic rendering by J. Marie Metz).

##### Etymology.

This species is named after Dr Jean Chazeau, for his gracious hospitality during the visits to New Caledonia by MEI.

##### Comments.

*Calophytuschazeaui* sp. nov. is known only from the southern province. It is often found associated with sclerophyll and maquis scrub habitats (e.g., Fig. 31A). Based on collecting records it is a more commonly encountered species relative to other members of the genus. GenBank sequences for this species (see Winterton et al. 2016: table S1): KT290077 (16S rDNA), KM884999 (28S rDNA), KM879116 (EF1a).

##### Specimens examined.

***Holotype*** male, New Caledonia: Province Sud: Chutes de Madeleine, Malaise trap (-22.226, 166.854), 28.XI.2000, J.H. Skevington (MEI123120, MNHN).

***Paratypes* .** New Caledonia: Province Sud: 1 males, 2 females, Parc Territorial de la Rivière, 23.5 km NNW Plum, Malaise trap [-22.049, 166.654], 213 m, 28.X–21.XI.2000, D.W. Webb, E.I. Schlinger, M.E. Irwin (MEI125567–69, CSCA); 1 male, Parc Territorial de la Rivière, 23.5 km NNW Plum, hand collected [-22.049, 166.654], 213 m, 28.XI.2000, D.W. Webb, E.I. Schlinger, M.E. Irwin, (MEI125566, CSCA); 14 males, Chutes de Madeleine, malaise trap (-22.226, 166.854), 28.XI.2000, J.H. Skevington (MEI123114–23, 123349, 123493–96, CNC); 5 males, Parc Territorial de la Rivière Bleue, Malaise trap (-22.100, 166.66), 28.XI.2000, J.H. Skevington (MEI123497–501, CSCA); 5 males, Plaine des Lacs, S of Grand Lac, hand netted, high shrub [-22.275, 166.900], 280 m, 14.X.1985, P. Bouchet (MEI028279–80, 30098, 30100–02, MNHN); 6 males, Rivière Bleue National Park, Malaise trap, (-22.251, 166.872), 20.IX–11.IX.2000, J. & A. Skevington (MEI125623–28, CNC); 1 male, 5 females, Rivière Bleue Provincial Park, 30 km NW Yate, Malaise trap, scrub on ridge [-22.117, 166.658], 310 m, 13.X–28.X.1986, L.B. de Larbogne, J. Chazeau, A. & S. Tillier (MEI030097, 030125–30, MNHN); 18 males, 16 females, Rivière Bleue Provincial Park, 25.8 km Rivière Bleue Road, Malaise trap across forest path [-22.11, 166.65], 213 m, various dates: 30.X–28.XI.1992, D.W. Webb, E.I. & M. Schlinger (MEI030103–17, 030122–23, 030138–9, 030141–54, CSCA); 5 males, 1 female, Rivière Bleue Provincial Park, Rivière Bleue, trail to Vallée de Pourina, Malaise trap across forest path, [-22.017, 166.733], 850 m, 19–28.XI.1992, D.W. Webb, E.I. & M. Schlinger, (MEI030118–21, 030133, 030132, CSCA); 3 males, 1 female, Upper Boulari River [La Coulee] [-22.180, 166.593], 17.XI.1968, C.R. Joyce, (MEI129018, 030096, 030099, 030127, BPBM); 1 female, Rivière Bleue Provincial Park, Rivière Bleue, Rivière Bleue Road, Malaise trap across forest path [-22.11, 166.65], 290 m, 16–19.XI.1992, D.W. Webb (MEI030156, CSCA); 3 females, Rivière Bleue Provincial Park, Rivière Bleue, 25.8 km Rivière Bleue Road, Malaise trap in maquis (scrub) [-22.117, 166.658], 290 m, various dates: 30.X–3.XI.1992, D.W. Webb, E.I. & M. Schlinger (MEI030134–6, CSCA); 9 females, Rivière Bleue Provincial Park, Rivière Bleue, 25.8 km Rivière Bleue Road Malaise trap across forest path, [-22.11, 166.65], 213 m, various dates: 5–28.XI.1992, D.W. Webb, E.I. & M. Schlinger, (MEI030131, 030137, 030140, 030148, 030150–54, CASC); 3 females, Rivière Bleue Malaise trap, scrub near ridge (maquis sur crête) [-22.098, 166.630], 310 m, 12–25.XI.1986, L.B. de Larbogne, J. Chazeau, A. & S. Tillier (MEI028277–8, 030124, MNHN).

#### Calophytus grandiosus sp. nov.

Taxon classificationAnimaliaTherevidaeAgapophytinae

4C34E496-5B60-5773-8ACE-5C90BB5CA68F

http://zoobank.org/1D84D7A3-0E7B-430A-9A05-C36101CB9AC8

Figs 2 , 5B , 6B , 7C , 11 , 12 , 22 , 31A , 32C

##### Diagnosis.

Black postocular setae scattered over occiput in both sexes; flagellum shorter than scape; scutum dark; wing infuscation as two bands, apex with white infuscation; costal cell hyaline except for brown infuscation distally; legs dark with white on dorsal surface of tibia; male abdomen lacking silver pubescence.

##### Description.

Length 10.0–12.5 mm. *Head*. Glossy black, smooth. Frons slightly verrucous, raised around base of antennae. Eyes separated by 4× width of median ocellus in both sexes. Parafacial silver pubescent, face glabrous medially. Occiput sparse silver pubescent, denser laterally and ventrally onto gena, admixed with scattered, black setae; posterior oral margin black, sparsely pubescent. Postocular macrosetae black scattered on occiput, not in distinct rows. Scape 1.3× head length, brown basally, black on apical 3/4, sparsely pubescent. Pedicel black, 1/10× length of scape, densely covered with short, black setae. Basal flagellomere ½× length of scape, short, black setae covering entire length. Second flagellomere subequal in length to third flagellomere, extremely short. Style minute. Palpus cylindrical basally, apex spatulate with a truncate tip; cream-coloured basally and medially towards apex, otherwise black. Mouthparts black except labellum, which is grey-brown; brown setose. *Thorax*. Dark brown. Matte black anteromedially on scutum. Silver pubescence on dorsal part of proepimeron, posterior katepisternum, lateral and extreme posterior part of scutum, scutellum, posterior anepimeron, metanepisternum, metepimeron, meron, and metakatepisternum; macrosetae black (np: 2, sa: 1, pa: 1, dc: 0, sc: 1); notum and katatergite with short, white setae. *Legs*. Black except white on dorsal surface of tibiae and basal 3/4 of mid- and hind basal tarsomere; sparsely silver pubescent, admixed with short, black setae. Fore- and midcoxa with four black anteroventral, marginal macrosetae. Hind coxa with anteroventral margin truncated with a single black, anteroventral, marginal macroseta. *Wing.* Strongly banded, dark areas infuscated with dark microtrichia; broad subapical band and a narrow band at mid length; apex with white infuscation; hyaline areas mostly lacking microtrichia. Pterostigma brown. Venation brown except anterior costal margin, basal Sc, and all veins at wing apex cream-coloured; cell m_3_ widely open at wing margin. Haltere stalk brownish, knob white. *Abdomen*. Segments mostly black, white laterally on tergite I and with a broad white band on segment II; shiny, lacking pubescence, sparsely covered with short, black setae; tergites I and II with long, white setae laterally. *Genitalia*. Male: tergite VIII with anterior margin slightly emarginated. Sternite VIII quadrate, wider posteriorly. Epandrium dark, quadrangular with posterolateral processes. Cercus broad, rounded posteriorly. Subepandrial sclerite wide, 2/3 width of epandrium; partially sclerotised, lateral margins more strongly so. Gonocoxites dark brown with dark, elongate, setae laterally, inner gonocoxal process curved medially along length, rounded apically; outer gonocoxal process angular. Aedeagus with dorsal apodeme of parameral sheath ‘T’-shaped, laterally forked apically; ventral apodeme divergently forked, arms broad; distiphallus robust, straight with numerous sinuous processes apically. Ejaculatory apodeme robust, roughly cylindrical, with a dorsal carina and anterior end with a blunt cap; lateral ejaculatory apodemes arc-like around basiphallus. Female: tergite VIII wider than long, narrower posteriorly, with a narrow anteromedial projection. Acanthophorite and acanthophorite spines dark brown. Sternite X triangular, anterior margin truncate. Spermathecal duct equal to 4 × length of furca. Spermathecal sac duct equal to 3.5 furcal lengths; sac ovoid, longer than wide; 0.5 × length of spermathecal sac duct.

**Figure 2.**
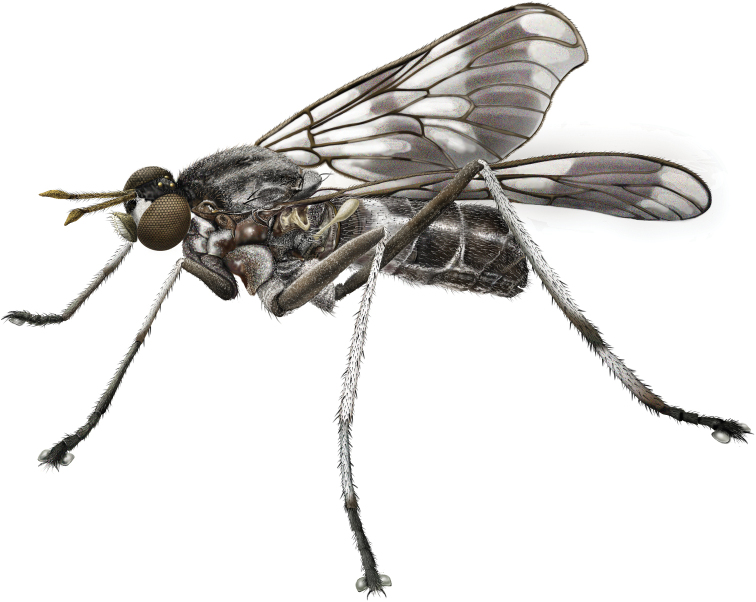
*Calophytusgrandiosus* sp. nov., male habitus (MEI030208) (artistic rendering by J. Marie Metz).

##### Etymology.

The species epithet is derived from the Latin *grandis*, great, large, magnificent, and -*osus*, fullness, abundance; referring to the distinctive size and striking wing patterning of this species. Gender is masculine.

##### Comments.

*Calophytusgrandiosus* sp. nov. is a relatively large species with distinctive wing banding and black and white legs. It is distinct from other *Calophytus* species and is rather isolated in the genus as sister to the rest of the genus (Fig. 4). This species is found in the mid- to upper-elevation forests (Figs 31, 32). GenBank sequences for this species (see Winterton et al. 2016: table S1): KT290078 (16S rDNA), KM885004 (28S rDNA), KM879121 (EF1a).

##### Specimens examined.

***Holotype*** male, New Caledonia: Province Sud: Sarraméa, Malaise trap in forest [-21.641, 165.846], 780 m, 2–5.XII.2001, T. Pape, B. Viklund (MEI135021, MNHN).

***Paratypes***. New Caledonia: Province Sud: female, 17 km NNE Nouméa, Mount Khogis, Malaise trap across path in rainforest [-22.176, 166.505], 425 m, 26.I.1996, M.E. Irwin, D.W. Webb, E.I. Schlinger (MEI071889, CSCA); female, 17 km NNE Nouméa, Mt. Koghis, Malaise trap [-22.167, 166.533], 500 m, 23–26.XII.1991, M.E. Irwin, D.W. Webb, (MEI030209, CSCA); 2 females, Rivière Bleue Provincial Park, 30 km NW Yaté, Station Parc 5, Malaise trap [-22.117, 166.658], 19.XI–4.XII.1985 & 20.XII.1985–8.I.1986, L.B. de Larbogne, J. Chazeau (MEI030210–11, MNHN); female, Mt. Koghis, Malaise trap in forest [-22.167, 166.533], 600 m, 2–5.XII.2001, T. Pape, B. Viklund (MEI135022, NHRS); female, Rivière Bleue Provincial Park, 30 km NW Yaté, Malaise trap across forest path [-22.117, 166.658], 27–28.XII.1991, M.E. Irwin, D.W. Webb (MEI030208, CSCA); female, 9.3 km NW Sarraméa, Malaise trap [-21.581, 165.787], 497 m, 17–24.XI.1998, M.E. Irwin, E.I. & M.B. Schlinger (MEI011284, CSCA); female, Rivière Bleue Provincial Park, trail to Upper Rivière Bleue, Malaise trap across forest path [-22.117, 166.658], 290 m, 19–28.XI.1992, D.W. Webb (MEI030212, CSCA). Province Nord: female, 5 km WSW Puébo [Pouébo], Mount Mandjélia, Malaise trap [-20.397, 164.528], 720 m, 27.VI–8.VII.2000, M.E. Irwin, E.I. Schlinger, D.W. Webb (MEI131366, CSCA).

#### Calophytus matilei sp. nov.

Taxon classificationAnimaliaTherevidaeAgapophytinae

55944B60-53F1-5637-B74E-DDAF1D71A3FE

http://zoobank.org/3C325DB8-82AC-4230-80C4-60D0583837D3

**Figure 7.**
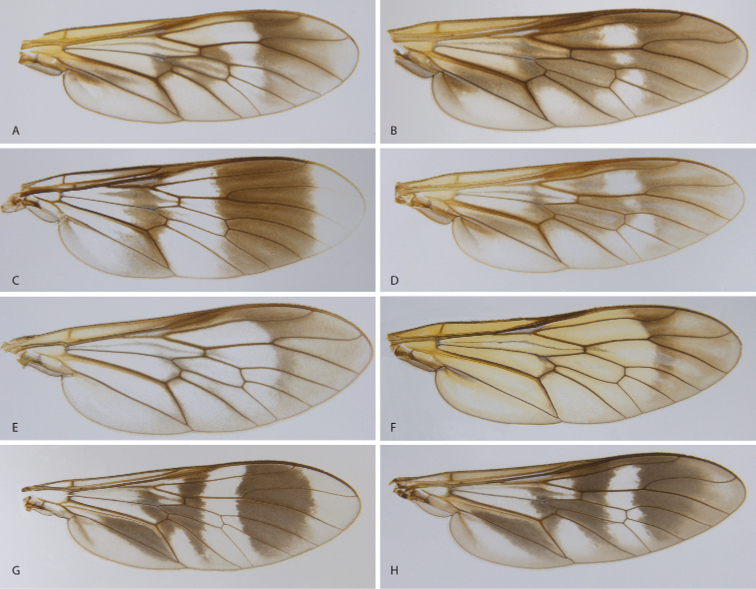
Wings of *Calophytus* gen. nov. and *Jeanchazeauia* gen. nov. **A***C.chazeaui* sp. nov. **B***C.schlingeri* sp. nov. **C***C.grandiosus* sp. nov. **D***C.matilei* sp. nov. **E***C.webbi* sp. nov. **F***C.monteithi* sp. nov. **G***J.amoa* sp. nov. **H***J.nubilosus* sp. nov. (female) (figures not to scale).

Figs 5D , 6C , 7D , 13 , 14 , 22

##### Diagnosis.

Black postocular setae as single row dorsally; flagellum shorter than scape; scutum dark with yellow along notopleural callus; legs yellow, mid- and hind coxae extensively yellow; wing with extensive infuscation, fenestrate in apical half; abdomen dark, male with silver velutum pubescence.

##### Description.

Length 7.8–10.6 mm. *Head*. Glossy black. Frons smooth, raised around base of antenna, sometimes with patch of silver pubescence lateral to and above base of antennae. Eyes separated by slightly more than 2× width of median ocellus. Occiput black with silver or gold pubescence laterally, extending ventrally onto gena, admixed laterally and ventrally with light brown to white, fine setae; parafacial silver pubescent dorsally, black pubescent ventrally; posterior buccal cavity brown, sparsely pubescent. Postocular macrosetae on occiput few in number, arranged dorsally in single row in both sexes, typically black, sometimes with orange suffusion. Scape 0.8× head length; yellow basally, black apically. Pedicel short, 1/8 length of scape, black, sparsely yellow pubescent, with numerous short, brown setae. Basal flagellomere ½× length of scape; elongate, gradually tapering to a blunt point apically with short, black setae covering entire length. Second flagellomere cylindrical. Third flagellomere subequal in length of second. Style subequal in length to third flagellomere, spiculate. Palpus more or less cylindrical, slightly capitate at apex; yellow basally, black at apex; yellow pubescent basally, black pubescent at apex; yellow setose basally, black setose at apex. Mouthparts yellow. *Thorax*. Dark brown, except prothorax, postpronotal lobe, and notopleuron yellow; prosternum, ventral proepimeron, posterodorsal katepisternum, posterior scutum, scutellum, subscutellum, metanepisternum, metepimeron, meron and metakatepisternum with silver pubescence; pronotum, anatergite, and dorsal proepimeron gold-yellow pubescent; macrosetae black (np: 2; sa: 1; pa: 1, dc: 0, sc: 1); scutum with relatively short setae. *Legs*. Yellow, except for black suffusion on foretarsus and hind coxa, legs with pale setae except dark setae apically on femora and on all tarsi; coxae silver pubescent, especially on hind coxa; hind femur with posteroventral macroseta yellow; hind basitarsomere with admix of white and gold setae ventrally and medially. Coxae with yellow setae anteriorly, hind coxa with two black setae laterally. Forecoxa with four marginal and one submarginal anteroventral, black and yellow macrosetae. Midcoxa with two marginal and three submarginal, anteroventral, black and yellow macrosetae. Hind coxa with anteroventral margin extended ventrally in a short point with three marginal, black or yellow macrosetae. *Wing*. Strongly marked, slightly variable and irregular amongst individuals. Costal cell with slight yellow infuscation; darker brown in cells sc, r adjacent to pterostigma, basal portions of r_1_ and r_2+3_, and r_4+5_, d, bm, basal portions of m_3_ and cua_1_, cup, and entire wing apex; infuscated areas also with dark microtrichia, hyaline areas of membrane largely void of microtrichia; pterostigma brown. Venation brown, except costa and R at base of wing gold; m_3_ widely open at wing margin. Haltere yellow. *Abdomen*. Segments mostly dark brown, males with tergites I–III and sternites II–V with portion of medial area lighter, light brown to dark yellow; female usually uniform dark brown although sometimes with lighter brown to dark yellow areas laterally on tergite II; male with tergites II–VI with silver velutum pubescence; tergites I–III with sparse short, pale, fine setae, longer laterally; tergite IV admixed with gold and black setae; remaining tergites with black setae. *Genitalia*. Male: epandrium narrowed posteriorly, with uniform scattered, dark setae. Cercus broad, truncate posteriorly. Subepandrial sclerite narrow, 1/3 width of epandrium; partially sclerotised, lateral margins more strongly so. Gonocoxites with scattered elongate brown setae; slightly denser ventrally; outer gonocoxal process sub-triangular in profile; inner gonocoxal process uniformly narrow, curved medially. Parameral sheath of aedeagus with ‘T’-shaped dorsal apodeme, arms slightly bifurcated; ventral apodeme fork relatively short, lobes rounded, distiphallus straight; basiphallus bulbous, lateral ejaculatory apodeme narrow, band-like with lateral process; Ejaculatory apodeme robust. Female: tergite VIII wider than long; anteromedial, projection long and narrow, 1/3× length of tergite VIII; posterior margin emarginated. Acanthophorite brown; acanthophorite spines brown admixed with short, brown setae. Sternite X sharply acute posteriorly. Spermathecal duct equal to four furcal lengths. Spermathecal sac duct equal to two furcal lengths; spermathecal sac ovoid, longer than wide; 1.5× length of spermathecal sac duct.

##### Etymology.

*Calophytusmatilei* sp. nov. is named in honour of the late Dr Loïc Matile, former curator of Diptera, Muséum national d’Histoire naturelle, Paris, France, who was extremely helpful to this project by providing loans of New Caledonian Therevidae.

##### Comments.

*Calophytusmatilei* sp. nov. is very similar to *C.schlingeri* sp. nov. but can differentiated based on the pattern of silver pubescence on the face, pattern of wing infuscation and whether the mid- and hind coxae are largely dark yellow or brown. These are the only known species in the genus with silver velutum pubescence on the male abdomen. *Calophytusmatilei* sp. nov. is known from forest habitats in the northern parts of the South Province. GenBank sequences for this species (see Winterton et al. 2016: table S1): KT290079 (16S rDNA), KM885000 (28S rDNA), KM879118 (EF1a).

##### Specimens examined.

***Holotype*** male, New Caledonia: Province Sud: Sarraméa, Malaise trap in forest [-21.641, 165.846], 780 m, 2–5.XII.2001, T. Pape, B. Viklund (MEI135020, MNHN).

***Paratypes***. New Caledonia: Province Sud: 6 males, 1 female, Sarraméa, Malaise trap in forest [-21.641, 165.846], 780 m, 2–5.XII.2001, T. Pape, B. Viklund (MEI135015–19, 135054–5, NHRS, CSCA); 5 females, Reserve Col d’Amieu, 7.5 km NW Sarraméa, Malaise trap [-21.585, 165.819], 300 m, 4–9.XI.2000, M.E. Irwin, E.I. Schlinger, D.W. Webb, (MEI123351–5, NHRS, CSCA); 4 females, Pointe du Cagou, base de Naemeni, humid forest on peridotite [-21.681, 166.335], 30 m, 5–8.XI.1984, Tillier, Bouchet (MEI030190–2, MNHN).

#### Calophytus monteithi sp. nov.

Taxon classificationAnimaliaTherevidaeAgapophytinae

79F34EBB-DCA2-57B0-B2F0-50EA81E42307

http://zoobank.org/5801CAFA-7A24-4293-BF56-FE445D34F1F6

Figs 7F , 15 , 22 , 32A

##### Diagnosis.

Body mostly dark yellow to orange; single row of orange postocular setae dorsally; flagellum shorter than scape; wing with pale yellowish infuscation, darker apically with hyaline fenestrations in most cells; head yellow except for black on upper frons and dorsolaterally on occiput; legs yellow except for dark tarsi apically.

##### Description.

Length: 10.3 mm. *Head*. Glossy dark yellow, upper frons and dorsolateral portion of occiput dull black. Frons smooth, raised around base of antenna, eyes separated by 3× width of median ocellus. Lateral occiput, lower frons, face, parafacial, and gena dull orange, sparsely pubescent; lateral and ventral occiput silver pubescent with scattered yellow setae ventrally; posterior oral margin orange, not pubescent. Postocular macrosetae yellow-orange, single row with a few scattered macrosetae ventral to the dorsal row. Scape ¾× head length; orange basally, dark brown on apical 1/4 sparsely yellow pubescent. Pedicel dark brown, sparsely yellow pubescent, with one whorl of short, black, setae with a few additional setae. Basal flagellomere 0.7× length of scape; gradually tapering to a blunt point apically; short, fine, black setae dorsally at base. Second flagellomere slightly conical, apex narrower than base; < 1/10× length of basal flagellomere. Third flagellomere 1/3× length of second flagellomere. Style small, spiculate. Palpus cylindrical, apex slightly capitate; orange; yellow pubescent admixed with yellow setae. Mouth parts orange with yellow setae. *Thorax*. Thorax dark yellow-orange; posterodorsal katepisternum, posterior metepimeron, anteroventral portion of meron, and posterodorsal metakatepisternum silver pubescent; macrosetae black (np: 2, sa: 1, pa: 1, dc: 0, sc: 1); scutum with short, black setae; katatergite, lateral postpronotum, postpronotal lobe, cervical sclerite, proepisternum, and lateral prosternum with short, yellow-white setae. Scutum with dark brown, dorsocentral stripes, narrow anteriorly, widening and merging into a single broad stripe posteriorly. *Legs*. Yellow except with small black spot ventrally at junction of femora and trochanters; base of hind femur and tibia and all tarsomeres with brown suffusion; legs sparsely yellow pubescent, admixed with short pale setae; dorsal 1/4 of hind coxa silver pubescent; all coxae with short, yellow setae. Forecoxa with two, midcoxa with four, and hind coxa with two yellow, anteroventral, marginal macrosetae. *Wing*. Membrane mostly hyaline with extensive pale yellow infuscation, most cells with central hyaline fenestration, wing apex with darker infuscation; membrane mostly lacking dark microtrichia, present as apical band, patch at apex of cell d, as elongate narrow stripe in cell br, and along all veins; pterostigma light brown. Cell m_3_ widely open at wing margin. Haltere stalk yellow, knob brown. *Abdomen*. Uniform dark yellow to orange, overlain with yellow-gold pubescence and fine pale setae, longer laterally; tergites I–IV with at least some short, black, setae medially. *Genitalia*. Not dissected.

**Figure 8.**
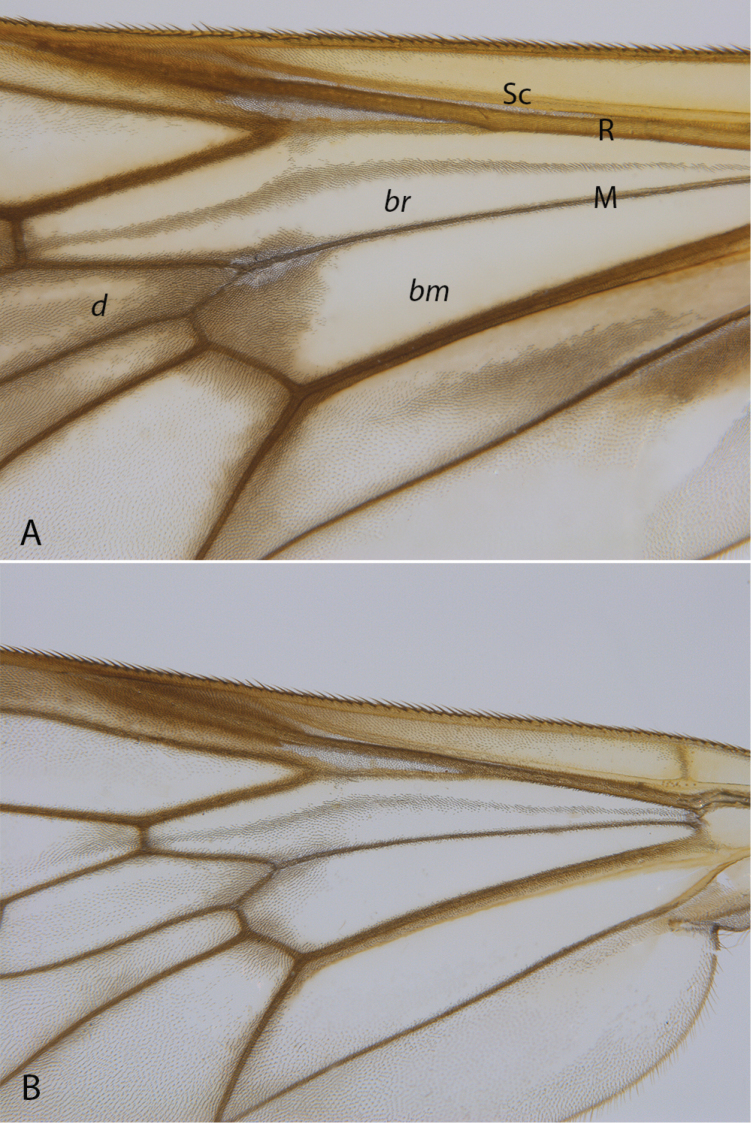
*Calophytus* spp. Wing detail showing cell *br* bisected with stripe of dark microtrichia along wing fold **A***C.chazeaui* sp. nov. **B***C.webbi* sp. nov.

##### Etymology.

This species is named in honour of Dr Geoffrey Monteith, Emeritus Senior Curator, Queensland Museum, Brisbane, who was one of the collectors of one of the two known female specimens of this species.

**Figure 9.**
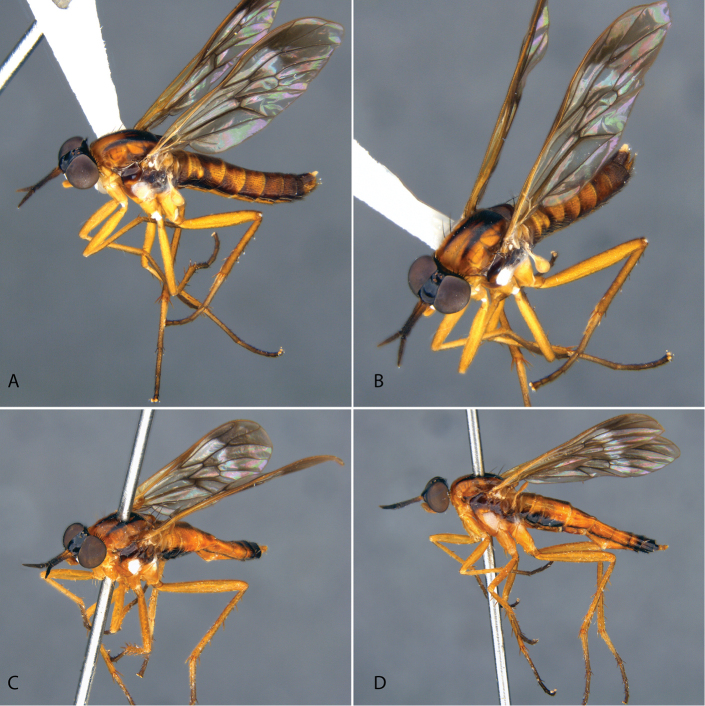
*Calophytuschazeaui* sp. nov. **A** adult male (MEI125624), lateral view **B** same, oblique view **C** adult female (MEI030131), oblique view **D** same, lateral view. Body length: male: 8.3 mm; female: 9.0 mm.

##### Comments.

*Calophytusmonteithi* sp. nov. is known only from two female specimens but can readily be distinguished from other species of *Calophytus* by the body being mostly orange except for the dark dorsocentral stripe posteriorly, dark tarsi distally, and the mostly hyaline wing with faint yellowish infuscation. This species is known from misty rainforest habitats at higher elevations (430–850 m) (Fig. 32A). The male is unknown.

**Figure 10.**
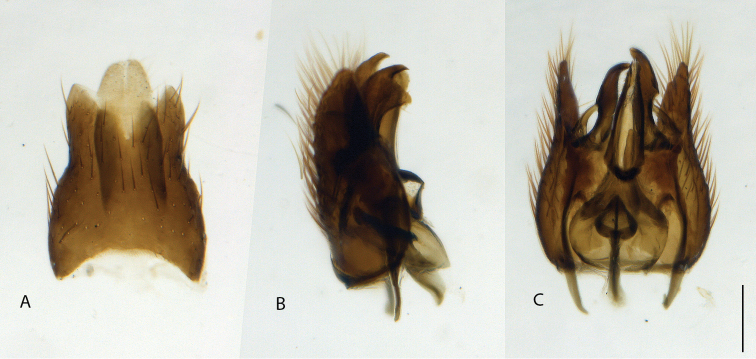
*Calophytuschazeaui* sp. nov., cleared male genitalia **A** epandrium **B** gonocoxites with aedeagus, lateral view **C** same, dorsal view with epandrium removed. Scale bar: 0.2 mm.

##### Specimens examined.

***Holotype*** female, New Caledonia: Province Sud: Col d’Amieu, humid forest, 430 m, 7.X.1984, 21°36'00"S, 165°48'08"E [-21.6, 165.802], Tillier & Bouchet, Collection #116a (MEI030193, MNHN).

**Figure 11.**
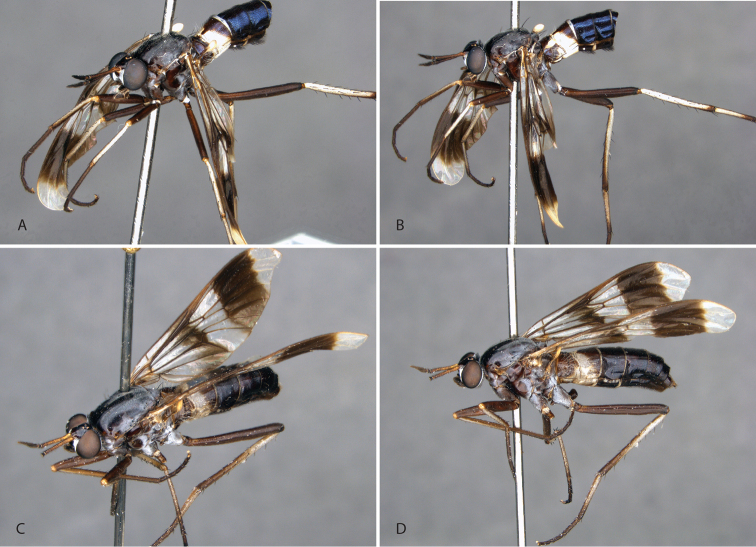
*Calophytusgrandiosus* sp. nov. **A** adult male (MEI135021), oblique view **B** same, lateral view **C** adult female (MEI030208), oblique view **D** same, lateral view. Body length: male: 10.3 mm [intact length; male terminal segments and genitalia removed]; female: 11.8 mm.

***Paratype***. New Caledonia: Province Nord: 1 female, 8711, 21°11'S, 165°18'E [-21.183, 165.3], 850 m, Aoupinie, top camp, 3–23.X.2001, C. Burwell & G. Monteith, Malaise, rainforest (MEI138464, QM).

**Figure 12.**
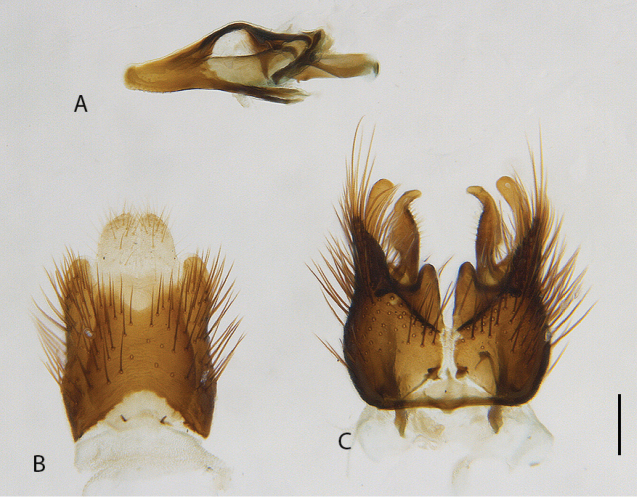
*Calophytusgrandiosus* sp. nov., cleared male genitalia **A** aedeagus, lateral view **B** epandrium **C** gonocoxites, ventral view. Scale bar: 0.2 mm.

#### Calophytus schlingeri sp. nov.

Taxon classificationAnimaliaTherevidaeAgapophytinae

21FE0300-21BE-59BC-B84E-85A8F1669B25

http://zoobank.org/76F032BE-4F50-46B1-908E-146A0BB5E1EE

Figs 5C , 6A , 7B , 16 , 17 , 18 , 22 , 23A , 31A

##### Diagnosis.

Flagellum shorter than scape; single row of postocular setae dorsally; scutum dark except for orange suffusion on notopleural callus; legs yellow except for tarsi dark apically; wing with dark infuscation, fenestrate apically; male abdomen with silver velutum pubescence.

##### Description.

Length 8.5–10.7 mm. *Head*. Glossy black. Frons smooth, raised around base of antenna. Eyes separated by slightly more than 2× width of median ocellus. Occiput black with silver or gold pubescence laterally, extending ventrally onto gena, admixed laterally and ventrally with light brown to white, fine setae; parafacial silver pubescent as elongate wedge shape, extending ventrally along margin of buccal area. Postocular macrosetae on occiput few in number, arranged dorsally in single row in both sexes, typically black, sometimes with orange suffusion. Scape 0.8× head length; yellow basally, black apically. Pedicel short, 1/8 length of scape, black, sparsely yellow pubescent, with numerous short, brown setae. Basal flagellomere ½× length of scape; elongate, gradually tapering to a blunt point apically with short, black setae covering entire length. Second flagellomere cylindrical. Third flagellomere subequal in length of second. Style subequal in length to third flagellomere, spiculate. Palpus more or less cylindrical, slightly capitate at apex; yellow basally, black at apex; yellow pubescent basally, black pubescent at apex; yellow setose basally, black setose at apex. Mouthparts yellow. *Thorax*. Dark brown to black, except prothorax, postpronotal lobe, and notopleuron yellow; prosternum, ventral proepimeron, posterodorsal katepisternum, posterior scutum, scutellum, subscutellum, metanepisternum, metepimeron, meron and metakatepisternum overlain with silver pubescence; pronotum, anatergite, and dorsal proepimeron gold-yellow pubescent; scutum with faint dorsocentral silver pubescent stripes; macrosetae black (np: 2; sa: 1; pa: 1, dc: 0, sc: 1); scutum with relatively short setae. *Legs*. Yellow, except for black suffusion on foretarsus and hind coxa, legs with pale setae except dark setae apically on femora and on all tarsi; coxae silver pubescent, especially on hind coxa; hind femur with posteroventral macroseta yellow; hind basal tarsomere with admix of white and gold setae ventrally and medially. Coxae with yellow setae anteriorly, hind coxa with two black lateral setae. Forecoxa with four marginal and one submarginal anteroventral, black and yellow macrosetae. Midcoxa with two marginal and three submarginal, anteroventral, black and yellow macrosetae. Hind coxa with anteroventral margin extended ventrally in a short point with three marginal, black or yellow macrosetae. *Wing*. Strongly marked, variable in shape, irregular. Costal cell with pale yellow infuscation; brown infuscation in cells sc, r adjacent to pterostigma, basal portions of r_1_ and r_2+3_, and r_4+5_, d, bm, basal portions of m_3_ and cua_1_, cup, and entire wing apex; infuscated areas also with dark microtrichia, hyaline areas of membrane largely void of microtrichia; pterostigma brown. Venation brown, except costa and R at base of wing gold; cell m_3_ widely open at wing margin. Haltere yellow. *Abdomen*. Segments mostly dark brown, males with tergites I–III and sternites II–V with portion of medial area lighter, light brown to dark yellow; female usually uniform dark brown although sometimes with lighter brown to dark yellow areas laterally on tergite II; male with tergites II–VI with silver velutum pubescence; tergites I–III with sparse short, pale, fine setae, longer laterally; tergite IV admixed with gold and black setae; remaining tergites with black setae. *Genitalia*. Male: epandrium narrowed posteriorly, with uniform scattered, dark setae. Cercus relatively narrow, rounded posteriorly. Subepandrial sclerite narrow, 1/3 width of epandrium; partially sclerotised, lateral margins more strongly so. Gonocoxites with scattered elongate brown setae; slightly denser ventrally; outer gonocoxal process sub-triangular in profile; inner gonocoxal process uniformly narrow, curved medially, spatulate apically. Parameral sheath of aedeagus with ‘T’-shaped dorsal apodeme, arms slightly bifurcated, ventral apodeme fork relatively short, lobes rounded, distiphallus straight; basiphallus bulbous, lateral ejaculatory apodeme narrow, band-like with lateral process; Ejaculatory apodeme robust. Female: tergite VIII wider than long; anteromedial, projection long and narrow, 1/3× length of tergite VIII; posterior margin emarginated. Acanthophorite brown; acanthophorite spines brown admixed with short, brown setae. Sternite X sharply acute posteriorly. Spermathecal duct equal to four furcal lengths. Spermathecal sac duct equal to two furcal lengths; spermathecal sac ovoid, longer than wide; 1.5× length of spermathecal sac duct. *Variation*. Some paratypes with frons sparsely pubescent; ventral parafacial variably silver and/or gold pubescent; all postocular macrosetae black; additional postocular macrosetae ventral to single dorsal row; apical 3/4 scape brown; basal flagellomere with covering of short, black, filiform setae variable from basal 1/4 to entire; notopleuron variably coloured light to dark brown; midcoxae variably gold to brown; coxae with anteroventral, marginal macrosetae number variable from 3–5 and variably coloured gold to black; hind coxa lateral macrosetae number variable, 0–2; wing infuscation variable in intensity and distribution.

##### Etymology.

Named in honour of the late Dr Evert I. Schlinger, who participated in the New Caledonian expeditions, from which much of the material for this paper was collected.

##### Comments.

*Calophytusschlingeri* sp. nov. is very similar to *C.matilei* sp. nov. and can be distinguished based on the pattern of silver pubescence on the face, pattern of wing infuscation and whether the mid- and hind coxae are largely dark yellow or brown. These are the only known species in the genus with silver velutum pubescence on the male abdomen. Specimens of *Calophytusschlingeri* sp. nov. were collected in a variety of habitats including dry sclerophyll forest, rainforest and maquis scrub (Fig. 31A). GenBank sequences for this species (see Winterton et al. 2016: table S1): KM885001 (28S rDNA), KM879117 (EF1a).

##### Specimens examined.

***Holotype*** male, New Caledonia: Province Sud: Plaine des Lacs, 5 km E of Grand Lac, hand netted [-22.267, 166.967], 300 m, 22–25.I.1984, M. Pogue, M. Epstein (MEI030164, MNHN).

***Paratypes***. New Caledonia: Province Sud: 1 male, 2 females, Plaine des Lacs, 5 km E of Grand Lac, black light (UV) [-22.267, 166.967], 300 m, 22–25.I.1984, M. Pogue, M. Epstein (MEI030165, 03173–4, UMSP); 4 males, 3 females, Plaine des Lacs, 5 km E of Grand Lac, hand netted [-22.267, 166.967], 300 m, 22–25.I.1984, M. Pogue, M. Epstein, male, (MEI030161–3, 030166, 030175–7, FSCA, CASC); 1 female, Mt. Koghis, Malaise trap in forest [-22.167, 166.533], 600 m, 2–5.XII.2001, T. Pape, B. Viklund (MEI135023, NHRS); 1 male, Rivière des Pirogues black light (UV) [approximated as -22.275, 166.687], 7–9.II.1984, M. Pogue, M. Epstein (MEI030167, UMSP); 2 males, 4 females, Rivière Bleue Provincial Park, 25.8 km Rivière Bleue Road, Malaise trap across forest path [-22.117, 166.658], 213 m, various dates: 5–28.XI.1992, D.W. Webb, E.I. & M. Schlinger (MEI030158–9, 030170–2, CSCA); 1 male, Rivière Bleue Provincial Park, Rivière Bleue Road, Malaise trap across forest path [-22.117, 166.658], 290 m, 5–16.XI.1992, D.W. Webb, male, (MEI030160, CSCA); 1 female, 17 km NNE Nouméa, Mt. Koghis, [Koghis stream waterfall] Malaise trap in tropical forest [-22.171, 166.512], 500 m, 21–29.XI.1992, D.W. Webb, (MEI030169, CSCA).

#### Calophytus webbi sp. nov.

Taxon classificationAnimaliaTherevidaeAgapophytinae

D56A0CCF-55BB-5AAC-B209-1BD6B218FF94

http://zoobank.org/9CD4647C-D573-4A06-B7A2-DB2E3EED144C

Figs 5E , 6D , 7E , 8B , 19 , 20 , 21 , 22 , 31B

##### Diagnosis.

Flagellum longer than scape; single row of black postocular macrosetae dorsally; body predominantly yellow; wing mostly hyaline, dark apically; scutellum overlain with sparse silver pubescence; abdomen yellow, tergites brown posteriorly; legs yellow, tarsi brownish; male abdomen lacking silver pubescence.

##### Description.

Length 5.5–8.1 mm. *Head*. Glossy black, yellow around base of antenna. Frons glossy, smooth, slightly raised around base of antennae; eyes separated by 3× width of median ocellus in both sexes. Occiput black with silver pubescence laterally and ventrally, admixed with pale setae laterally and onto gena; single row of black to orange postocular macrosetae in both sexes. Parafacial dark, yellowish laterally, overlain with silver pubescence along eye margin; posterior oral margin yellow, not pubescent. Scape 3/4 of head length, dark yellow, brown suffusion apically. Basal flagellomere 2.5× length of scape, cylindrical, abruptly tapering to a blunt point apically, densely covered with brown pubescence. Second flagellomere subapical, subequal in length to third flagellomere; style small, spiculate. Palpus cylindrical, apex slightly capitate. Mouthparts yellow with pale setal pile, yellow on labium, black on labellum. *Thorax*. Yellow; scutum with dark dorsocentral stripes, narrow anteriorly, thickening posteriorly and merging medially into a single stripe before posterior margin, brownish suffusion sometimes posteriorly on scutum; scutellum brown dorsally; posterodorsal katepisternum, metanepisternum, metepimeron, and metakatepisternum overlain with silver pubescence; sparse silver pubescent on posterior scutum and scutellum; macrosetae black (np: 1, sa: 1, pa: 1, dc: 0, sc: 1); scutum, katatergite, lateral postpronotum, postpronotal lobe, cervical sclerite, proepisternum, and lateral prosternum with short, yellow setae. *Legs*. Yellow except with small black spot ventrally at union of femora and trochanters; foretarsus light brown; sparsely pubescent, dorsal part of hind coxa silver pubescent; legs with short, yellowish, setae, except foretarsus with brown setae. Forecoxa with some longer, gold, filiform setae anteriorly. Forecoxa with two macrosetae; mid- and hind coxae with three anteroventral macrosetae. *Wing*. Membrane mostly hyaline; costal, subcostal, pterostigma and adjacent r cells, and entire wing apex light brown infuscation; membrane with extensive areas bare of microtrichia. Venation brown with dark microtrichia along veins, m_3_ widely open at wing margin. Haltere stalk yellow, knob light brown. *Abdomen*. Yellow, posterior and lateral margins of tergites II–VII brown, terminal segments darker; glossy, not pubescent and male lacking silver pubescence; sparse fine pale setae; medial setae darker, especially on terminal segments. *Genitalia*. Male: tergite VIII anterior margin slightly emarginate; laterally with brown setae. Cercus relatively narrow, rounded posteriorly. Sternite VIII quadrate, wider posteriorly; posterior 1/6 with brown setae. Epandrium yellow basolaterally and at extreme posterolateral margin, otherwise brown; brown setae sparsely distributed; subepandrial sclerite narrow, 1/3 width of epandrium, partially sclerotised, lateral margins more strongly so. Gonocoxites yellow-brown, elongate dark setae sparsely distributed, except lacking setae ventromedially; outer gonocoxal process sub-triangular; aedeagus with ejaculatory apodeme sub-cylindrical; inner gonocoxal process narrow; dorsal apodeme of parameral sheath ‘T’-shaped, ventral apodeme forked; lateral ejaculatory apodeme band-like with small outer process. Female: acanthophorite and acanthophorite spines yellow-brown. Tergite VIII wider than long, with a narrow anteromedial projection, posterior margin slightly emarginated. Sternite VIII gold; long, brown, setae sparsely distributed, bare laterally and more dense on posterior lobes. Median lobe of tergite IX reduced, very short. Sternite X quadrangular with short, brown setae. Spermathecal duct 5× furcal length. Spermathecal sac duct 3.5× furcal length; spermathecal sac ovoid, longer than wide; 0.5× length of spermathecal sac duct.

##### Etymology.

This species is named after the late Dr Donald W. Webb, who was a member of several of the expeditions to New Caledonia and was one of the collectors of this species.

##### Comments.

*Calophytuswebbi* sp. nov. is a relatively diminutive, bright yellow species with distinctive antennae that readily distinguishes it from all other *Calophytus* species (Figs 5E, 19, 20). This species appears endemic to the tropical dry forest of the Pindai Peninsula (Fig. 31B) on the west coast of the Northern Province (Fig. 22); it is only known from a series of specimens collected from two localities on this peninsula. Genbank sequences for this species (see Winterton et al. 2016: table S1): KT290080 (16S rDNA), KM885002 (28S rDNA), KM879120 (EF1a); KT306930 (CAD) (not KT306928 as reported in Winterton et al. 2016).

##### Specimens examined.

***Holotype*** male, New Caledonia: Province Nord: 45 m, Presqu’île de Pindaï, 2.5 km WSW Népouï, Malaise, “21.383°S, 164.974°E” [GPS coordinates apparently erroneous; approximated instead as -21.33, 164.96], 26.XI–4.XII.2000, M.E. Irwin, E.I. Schlinger, D.W. Webb (MEI123356, MNHN).

***Paratypes***. New Caledonia: Province Nord: 1 female, Presqu’île de Pindaï, 2.5 km WSW Népouï, Malaise, “21.383°S, 164.974°E” [GPS coordinates apparently erroneous; approximated instead as -21.33, 164.96], 10–17.XI.2000, M.E. Irwin, E.I. Schlinger, D.W. Webb (MEI108224, CSCA); 8 males, [Presqu’île de Pindaï], 3 km SW Népouï, Pindai Forest, Malaise trap in costal forest [-21.35, 164.964], 13–23.XI.1992, D.W. Webb, E.I. & M. Schlinger (MEI030180–7, CSCA); 3 males, [Presqu’île de Pindaï] 3 km SW Népouï, Pindai Forest, Malaise trap in coastal forest [-21.35, 164.964], 7–13.XI.1992, D.W. Webb, E.I. Schlinger, M. Schlinger (MEI030178-9, 030188, CSCA).

#### Jeanchazeauia gen. nov.

Taxon classificationAnimaliaTherevidaeAgapophytinae

086B4049-5361-5AC3-B9C8-7138B098AC09

http://zoobank.org/599FD78A-C5E9-417E-A18B-18937C798CF1

##### Type species.

*Jeanchazeauianubilosus* sp. nov., here designated.

##### Diagnosis.

Frons width slightly wider than ocellar tubercle with little sexual dimorphism; postocular macrosetae arranged in one row in both sexes; femoral velutum patches absent; hind femur with anteroventral macroseta(e) subapically, frequently as a row; hind femur with series of posteroventral macrosetae; cell *br* with scattered microtrichia; male gonocoxite lacking medial atrium and velutum patch; female tergite VII with broad anteromedial process; tergite VIII narrowly band-like and ‘T’-shaped.

##### Description.

Antennal length variable, longer than head to equal to head length; flagellum cylindrical, slightly tapered distally, length subequal to combined length of scape and pedicel; scape narrow, elongate (> 3× pedicel length); upper frons flat, slightly raised around base of antennae; head shape in profile higher than long; male frons width at narrowest point slightly wider than ocellar tubercle, little sexual dimorphism in frons width; parafacial without setae; postocular macrosetae arranged in one row immediately lateral of ocellar tubercle in both sexes; prosternum without setae; abdominal tergite II all setae uniform and regular in length; fore- and hind femoral velutum patches absent; fore- and midfemoral macrosetae absent; hind femur with anteroventral macroseta(e) subapically, frequently as a row; hind femur with posteroventral macrosetae short, present as series around middle of segment; posterior surface of midcoxa without setae; hind femur and tibia relatively long compared to other legs; post-spiracular setae absent. Scutal chaetotaxy (pairs): np, 1–2; sa, 1; pa, 1; dc, 0 or 1–2 (minute); sc, 1; wing cell m_3_ open, cell br with scattered microtrichia, not restricted to wing fold; uniform wing dark infuscation to strongly banded. Male genitalia with dorsal apodeme of aedeagus ‘T’-shaped, distiphallus narrow, ventral apodeme with forked arms divergent; gonocoxite with posteromedial margins proximal to each other, velutum pubescence absent, inner gonocoxal process present, ventral lobe small, usually rounded apically; female genitalia with tergite VII with broad anteromedial process; tergite VIII narrowly band-like and ‘T’-shaped, with narrow anteromedial process; female acanthophorite setae with two sets present (A1 & A2); A1 slender, elongate acuminate apically, A2 series slightly reduced in size; sternite VIII cup-like, extensively pilose, greatly emarginate posteromedially; spermathecal sac elongate and rounded apically, not lobed; three spermathecae joining to spermathecal duct near junction of bursa copulatrix.

##### Etymology.

This new genus is named in honour of Dr Jean Chazeau, who extended unbridled hospitality and assistance to the members of our expeditions during our stays in New Caledonia. Gender is masculine.

##### Comments.

Of the three species in this genus, the male is known only for *J.nubilosus* sp. nov., which exhibits distinct sexual dimorphism in wing patterning. The modification of both tergite VII and tergite VIII in the female appears unique to this genus amongst all Therevidae. Although members of some Xestomyzinae (e.g., *Lynborgiaammodyta* Irwin, 1973), Agapophytinae (e.g., *Agapophytusbicolor* (Kröber, 1928)) and Therevinae (e.g., *Apenniverpavenezuelensis* Webb, 2005) have a modified ‘T’-shaped female tergite VIII remarkably similar in shape to members of *Jeanchazeauia* gen. nov., in all cases tergite VII is not modified at all. Species in this genus are relatively rare compared to the more widely encountered *Calophytus* gen. nov. and appear to be more restricted to montane rainforest habitats (Figs 31, 32).

##### Included species.

*Jeanchazeauiaamoa* sp. nov., *J.nubilosus* sp. nov., and *J.rufinatus* sp. nov.

### Key to species of *Jeanchazeauia* gen. nov.

**Table:** 

1	Legs with white macrosetae (male unknown) (Fig. 24)	***J.amoa* sp. nov.**
–	Legs with dark macrosetae (Figs 25, 28)	**2**
2	Frons densely silver pubescent; abdomen with broad silver pubescent band anteriorly on tergite II; posterior margin of tergite III and all of tergites IV and V reddish-orange; scutum with broad, silver, dorsocentral stripes (male unknown) (Figs 28, 29)	***J.rufinatus* sp. nov.**
–	Frons with mostly dull black or brownish pubescence; abdomen entirely brown-black, silver velutum pubescence on tergites II–IV in male, present only along posterior margin of tergites I–IV in female; scutum sparsely pubescent, lacking dorsocentral stripes (Figs 25, 26)	***J.nubilosus* sp. nov.**

#### Jeanchazeauia amoa sp. nov.

Taxon classificationAnimaliaTherevidaeAgapophytinae

2C16259F-DC8B-5566-B36D-35FE39DF7145

http://zoobank.org/8A0B7D2E-0B4D-4AF4-A816-EE746682CDF7

Figs 7G , 23B , 24 , 30

##### Diagnosis.

White macrosetae on tibiae, in two rows on hind tibia; frons dark; abdomen mostly dark brown; wing banded, apex hyaline.

##### Description.

Length 7.8–9.8 mm. *Head*. Dark brown; mostly silver pubescent, upper frons slightly bronze pubescent, frons with short, black, setae on dorsal half; eyes separated by width of ocellar tubercle. Occiput flat, overlain with silver pubescence, matte black pubescence dorsally along postocular ridge; white, elongate setae ventrolaterally and onto gena; postocular macrosetae black, in a single row dorsally. Scape 0.3× head length; dark brown, silver pubescence admixed with short, black setae except on medial surface. Basal flagellomere 1.3× length of scape; elongate, tapering to a blunt point apically; sparsely silver pubescent with short, fine, black setae dorsally at base. Second flagellomere apical; slightly conical, apex narrower than base; < 1/10× length of basal flagellomere. Third flagellomere minute. Style small, spiculate. Palpus one segmented; cylindrical, apex slightly capitate; brown; silver pubescent admixed with white setae. Mouthparts brown with brown setae. *Thorax*. Dark brown, mostly sparsely silver pubescent; scutum with very faint grey pubescent dorsocentral stripes; scutellum black, matte pubescent dorsally, bronze and silver pubescent on posterior margin; anterior anepisternum and katepisternum and posterior anepimeron glossy, glabrous; macrosetae black (np: 1, sa: 1, pa: 1, dc: 0, sc: 1); scutum with short, black, setae medially, white laterally; postpronotum, postpronotal lobe, cervical sclerite, proepisternum, and lateral prosternum with short, white setae; katatergite admixed with erect white and black setae. *Legs*. Brown except for apical 1/2 of foretibia and base of all basitarsi white; sparsely silver pubescent; short, black setae where cuticle is brown, white setae where cuticle is white; all macrosetae white. Coxae admixed with long, black and white, filiform setae. Forecoxa with two, midcoxa with three, and hind coxa with four black or white, anteroventral, marginal macrosetae. Hind coxa with one black, lateral macroseta. Hind femur with two white subapical anteroventral macroseta; short series of minute, dark, posteroventral macrosetae barely evident along middle of femur. White macrosetae on mid and hind tibia; midtibia with few macrosetae; hind tibia with white macrosetae arranged in two dorsal rows, another row of 3–5 five macrosetae present anteroventrally. *Wing*. Membrane hyaline with two broad dark bands; basal band originating at pterostigma and covering membrane to posterior wing margin; apical band covering wing tip except apical 1/6 of wing; membrane completely covered with microtrichia. Pterostigma dark. Veins dark; cell m_3_ wide open at wing margin. Haltere stalk and base of knob dark brown, knob white apically. *Abdomen*. Dark brown, sparsely silver pubescent, sparsely brown pubescent medially on tergites I–III; covered with short, black, setae, apical segments with longer setae laterally; tergites I and II with long, white, setae laterally. *Genitalia*. Female: tergites VI–VIII modified with anterior margin with medial process, broad and barely evident in tergite VI, to greatly elongate and narrow in tergite VIII, tergite VII representing an intermediate between the two; tergite VIII much wider than long; dark elongate setae present laterally on tergites VI–VIII. Sternite VIII slightly longer than wide, convex ventrally, posterior lobes tapering sharply posteriorly, separated by distance 3/4 width of one lobe, with a medial aedeagal guide; extensive elongate, setae over much of surface, bare at extreme lateral margin. Acanthophorite dark brown; acanthophorite spines dark, six tapered spines in A1 series, A2 series spines indistinguishable in size and thickness from rest of acanthophorite setae. Sternite X quadrate, with posterolateral edges expanded laterally; posterior margin widely rounded; dark brown; short, brown setae. Furca longer than wide, semicircular anteriorly, tapered to a point posteriorly; spermathecal sac relatively small and elongate, spermathecal sac duct and spermathecal sac indistinguishable; spermathecal ducts origination on spermathecal sac duct immediately adjacent to furcal membrane.

##### Etymology.

Named for the mountain peak, Pic d’Amoa, the type locality for this species and is a noun in apposition.

##### Comments.

*Jeanchazeauiaamoa* sp. nov. is the only species in the genus *Jeanchazeauia* that has white macrosetae on the legs. It is otherwise a comparatively drab coloured species, being mostly dark brown. It is one of the few species of New Caledonia stiletto flies known only from the Northern Province. The male is unknown.

##### Specimens examined.

***Holotype*** female, New Caledonia: Province Nord: “8905” 20°58'S, 165°17'E, Malaise [approximated as -20.963, 165.277], 500 m, Pic d’ Amoa, N slopes, 24.XI.01–31.I.2002, Burwell, Monteith (MEI138463, MNHN).

***Paratype***. New Caledonia: 1 female, Province Nord: “Mt. Mandjanie”, 5.3 km WSW P[o]uebo [approximated as -20.402, 164.525], 9–26.XI.1992, D.W. Webb, 550 m, Malaise trap in tropical forest (MEI030205, CSCA).

#### Jeanchazeauia nubilosus sp. nov.

Taxon classificationAnimaliaTherevidaeAgapophytinae

9357196F-BE26-5A86-8402-E475EBF46982

http://zoobank.org/F826CA19-F56D-4391-A6CC-EAA198ECE40C

Figs 3 , 6E , 7H , 23C , 25 , 26 , 27A–F , 30 , 31A, C , 32B

##### Diagnosis.

Black macrosetae on legs; frons dark, sparsely pubescent; female abdomen dark with white bands; female wing with strong banding, irregular basally, wing apex dark; male wing with uniform infuscation.

##### Description.

Length 8.3–9.6 mm. *Head*. Black to brown; mostly silver pubescent, upper frons sparsely pubescent, admixed with black setae, longer in male, a few setae dorsolateral to antennal bases, eyes separated by width of ocellar tubercle in both sexes; parafacial black with silver pubescence. Occiput black, matte pubescent, more bronze coloured along postocular ridge, silver laterally and ventrally. Postocular macrosetae mostly black, in a single row in both sexes, multiple irregular rows laterally, replaced by white finer setae laterally and on gena. Scape ½× head length; dark brown, sparsely silver pubescent; long, black, fine filiform setose except medial surface bare of setae. Basal flagellomere ½× length of scape, elongate and gradually tapering to a blunt point apically; sparsely silver pubescent with short, fine, black setae around base. Second flagellomere slightly conical, apex narrower than base; < 1/10× length of basal flagellomere. Third flagellomere subequal in length to second, conical. Style small, spiculate. Palpus one segmented, cylindrical, dark brown basally, light brown apically; silver pubescent admixed with white, setae basally, brown apically. Mouthparts brown with brown setae. *Thorax*. Dark brown-black with black and silver pubescence; scutum brown pubescent with anterior postpronotal lobe and notopleuron sparsely silver pubescent; scutellum with black matte pubescence anteriorly and medially, bronze pubescent along posterior margin; pleuron silver pubescent with two brown glabrous vertical bands passing from the notopleuron at the prothoracic spiracle posteroventrally through the anterior katepisternum, and from the wing base posteroventrally through the meron; macrosetae black (np: 3, sa: 1, pa: 1, dc: 1, sc: 1); scutum with short, black setae; postpronotal lobe, cervical sclerite, proepisternum, and lateral prosternum with white setae; katatergite with mostly white setae, both sides with one dark brown seta. *Legs*. Dark brown; coxae with dense silver pubescence, otherwise sparsely pubescent and admixed with short, black setae; macrosetae mostly black with a few light brown macrosetae on hind tibia. Coxae with white setae; forecoxa with two, midcoxa with three, and hind coxa with five black, anteroventral, marginal macrosetae; hind coxa lacking lateral seta. Posterior surface of femora with long, white setae admixed with short, black setae. Hind femur with one or two subapical anteroventral macrosetae; series of minute posteroventral macrosetae barely evident along middle of femur. *Wing*. Male wing membrane with entirely grey infuscation, darker subapically near anterior margin and apices of cells br and bm; membrane completely covered with microtrichia. Female wing strongly banded, with extensive hyaline areas; pterostigma brown; venation brown, cell m_3_ widely open at wing margin. Haltere entirely dark brown, knob slightly lighter ventrally. *Abdomen*. Dark brown-black, short, black, setae on all segments, tergites I and II laterally and sternites I–V with long, white setae; male tergites II–IV and intersegmental membrane on sternites II and III covered with silver velutum pubescence. *Genitalia*. Male: tergite VIII emarginate medially; black setose laterally on posterior 1/2. Sternite VIII quadrate, slightly wider posteriorly, posterior margin black setose. Epandrium quadrate, emarginate anteriorly and posteriorly, lateral corners only slightly extended posteriorly; dark brown setae longer posterolaterally. Cerci bluntly pointed posteriorly, dark brown setae dorsally and apically. Subepandrial sclerite wide, 1/2 width of epandrium; partially sclerotised, lateral margins more strongly so. Gonocoxites rounded with subtriangular outer gonocoxal process; long, dark setae, densely spaced laterally and small patch ventromedially; inner gonocoxal process smoothly curving medially, acuminate, lacking setae apically; ventral lobe minutely setose ventrally, gonostylus curved dorsomedially with a broad, dorsal process at 2/3 of length with medial and lateral carinae basodorsally, dark brown setose ventrally and on medial face. Aedeagus with dorsal apodeme of parameral sheath ‘T’-shaped, narrow posteriorly and widened anteriorly with anterolateral corners curving ventrally and laterally; distiphallus wide basally with lateral carinae, narrow apically, distiphallus spinose laterally; ventral apodeme dorsoventrally flattened, bifurcations subparallel to ejaculatory apodeme; ejaculatory apodeme robust, roughly cylindrical; posterior end of ejaculatory apodeme broadened laterally and ventrally expanded to a point creating a basket-shaped posterior face, lateral ejaculatory apodeme short and subtriangular with lateral process; basiphallus membranous anteriorly. Female: tergites VII and VIII highly modified, much wider than long, with a long and narrow anteromedial process; posterior margin slightly emarginate; with elongate dark setae. Sternite VIII slightly longer than wide, convex ventrally, posterior lobes tapering sharply posteriorly, separate, with a median aedeagal guide; extensive elongate, orange setae, bare at extreme lateral margin. Median lobe of tergite IX reduced, very short and not sclerotised. Acanthophorite with acanthophorite spines dark brown, A2 series spines indistinguishable from rest of acanthophorite setae. Sternite X quadrate. Cercus slightly laterally flattened, extended posteriorly, longer than wide, membranous, slightly sclerotised; furca longer than wide, semicircular anteriorly, tapered to a point posteriorly; origin of spermathecal ducts occurring immediately adjacent to furcal membrane; three spermathecae, much longer than wide, saclike, wider apically than basally; spermathecal duct thickened basally, extremely narrowed basal to spermathecae; bifurcation of spermathecal sac duct with spermathecal ducts very close to furcal membrane; spermathecal sac short, 2.5× furcal lengths; sac ovoid, longer than wide; ¾× length of spermathecal sac duct.

**Figure 3.**
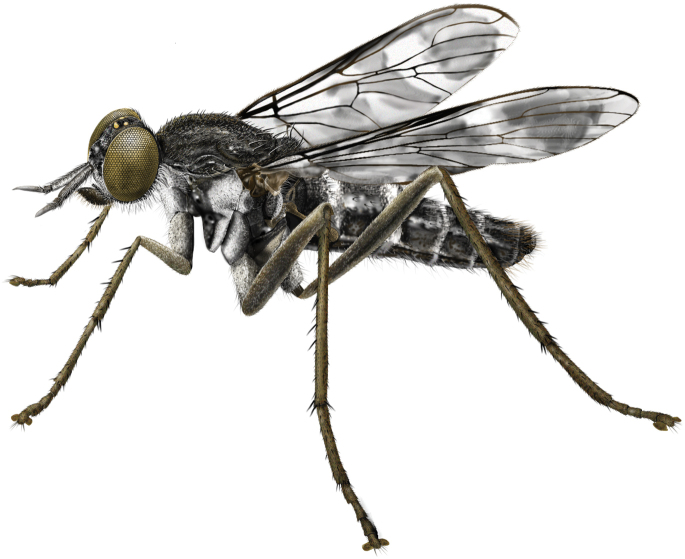
*Jeanchazeauianubilosus* sp. nov., female habitus (MEI030203) (artistic rendering by J. Marie Metz).

##### Etymology.

Derived from the Latin *nubilus*, cloudy, and -*osus*, full of; referring to the dark wing infuscation of the male.

**Figure 4.**
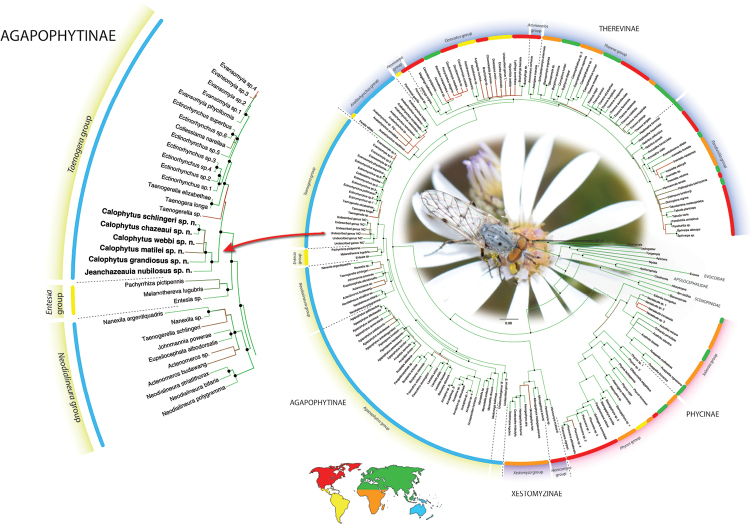
Phylogenetic placement of *Calophytus* gen. nov. and *Jeanchazeauia* gen. nov. in Agapophytinae based on supermatrix analysis of DNA sequence data. Figure modified after Winterton et al. (2016: fig. 3).

##### Comments.

*Jeanchazeauianubilosus* sp. nov. exhibits remarkable sexual dimorphism in wing pattern, but little to no other differences in the body or head. The male has largely uniform dark infuscate wings, while the wings of the female are strongly banded. It is not known if this condition extends to other species in the genus where the males are unknown, and such dramatic sexual dimorphism in wing patterning is rare in stiletto flies to this degree. *Jeanchazeauianubilosus* sp. nov. has been collected in dry to humid tropical forest at higher elevations on several mountains in New Caledonia (e.g., Figs 31A, C, 32B). GenBank sequences for this species (see Winterton et al. 2016: table S1): KM885007 (28S rDNA), KM879119 (EF1a).

**Figure 5.**
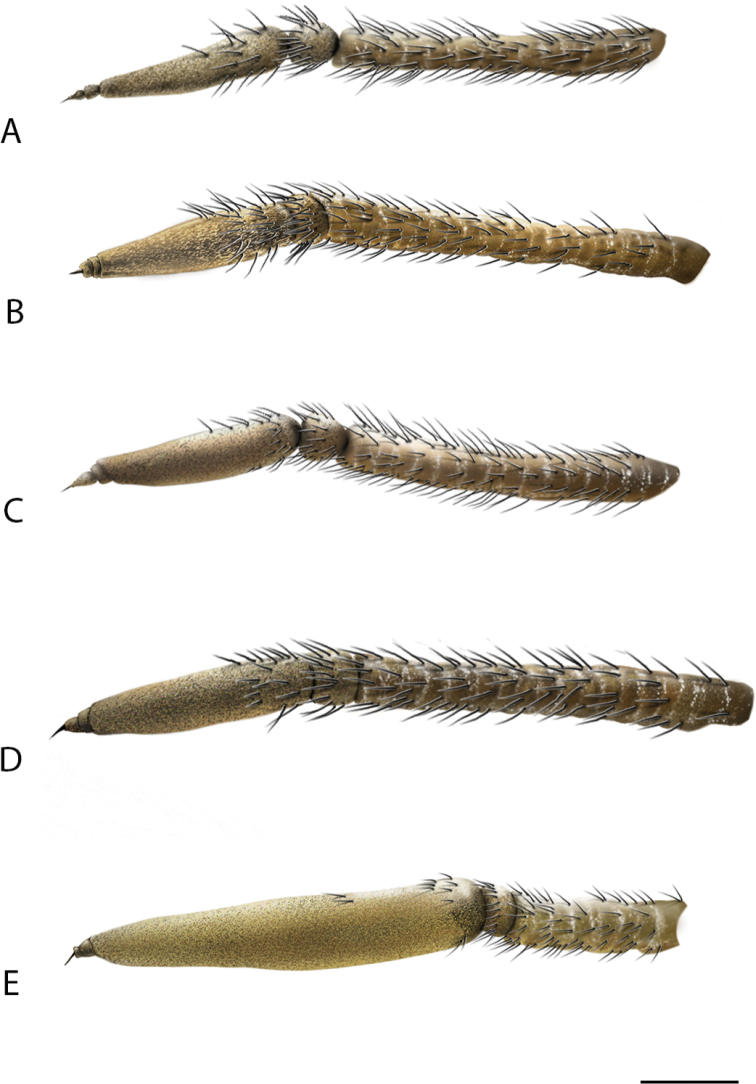
Lateral view of antennae of *Calophytus* gen. nov. **A***C.chazeaui* sp. nov. **B***C.grandiosus* sp. nov. **C***C.schlingeri* sp. nov. **D***C.matilei* sp. nov. **E***C.webbi* sp. nov. (figures not to scale) (drawings by J. Marie Metz).

##### Specimens examined.

***Holotype*** male, New Caledonia: Province Nord: Mt. Dzumac [Dumbea, approximated as -22.106, 166.458], black light (UV), 27–28.II.1984, M. Pogue, M. Epstein (MEI030206, MNHN).

**Figure 6.**
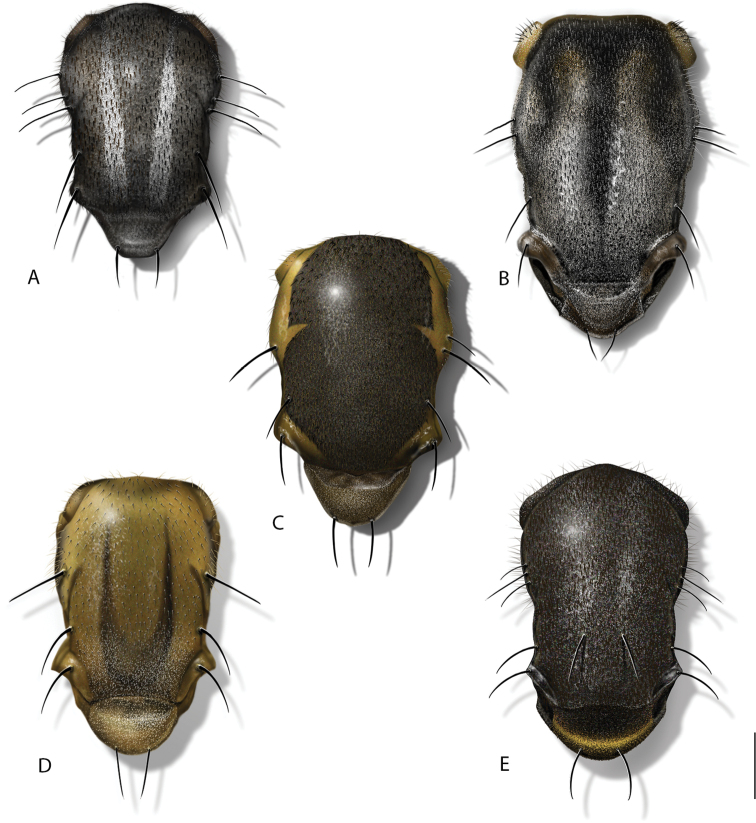
Scutum and scutellum of *Calophytus* gen. nov. and *Jeanchazeauia* gen. nov. **A***C.schlingeri* sp. nov. **B***C.grandiosus* sp. nov. **C***C.matilei* sp. nov. **D***C.webbi* sp. nov. **E***J.nubilosus* sp. nov. (figures not to scale) (drawings by J. Marie Metz).

**Figure 13.**
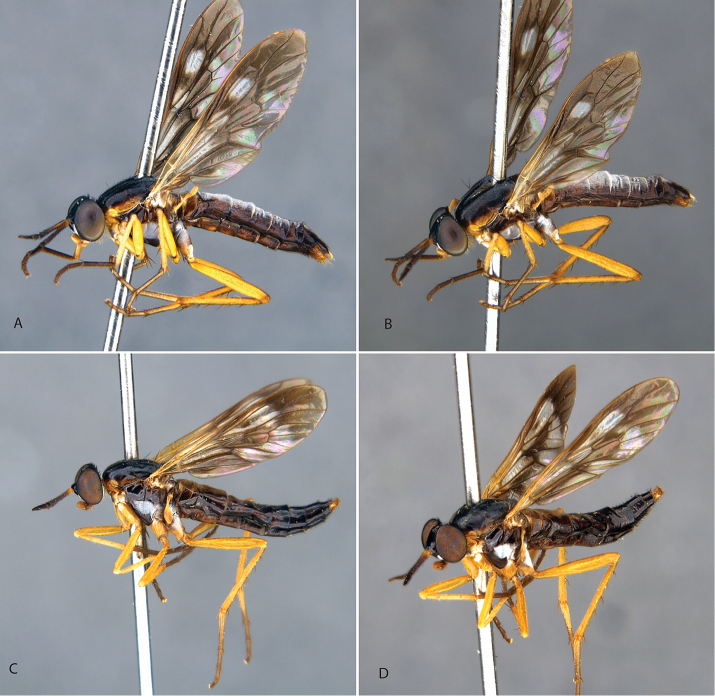
*Calophytusmatilei* sp. nov. **A** adult male (MEI135018), oblique view **B** same, lateral view **C** adult female (MEI071892), oblique view **D** same, lateral view. Body length: male: 8.0 mm; female: 9.4 mm.

**Figure 14.**
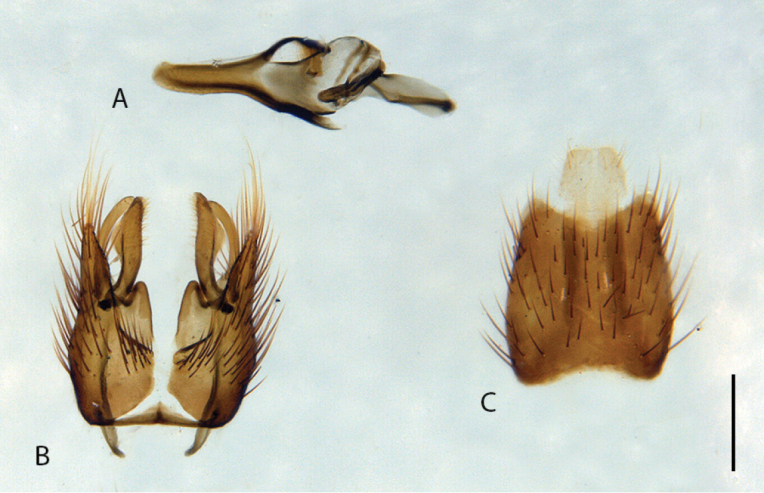
*Calophytusmatilei* sp. nov., cleared male genitalia **A** aedeagus, lateral view **B** gonocoxites, ventral view **C** epandrium. Scale bar: 0.2 mm.

**Figure 15.**
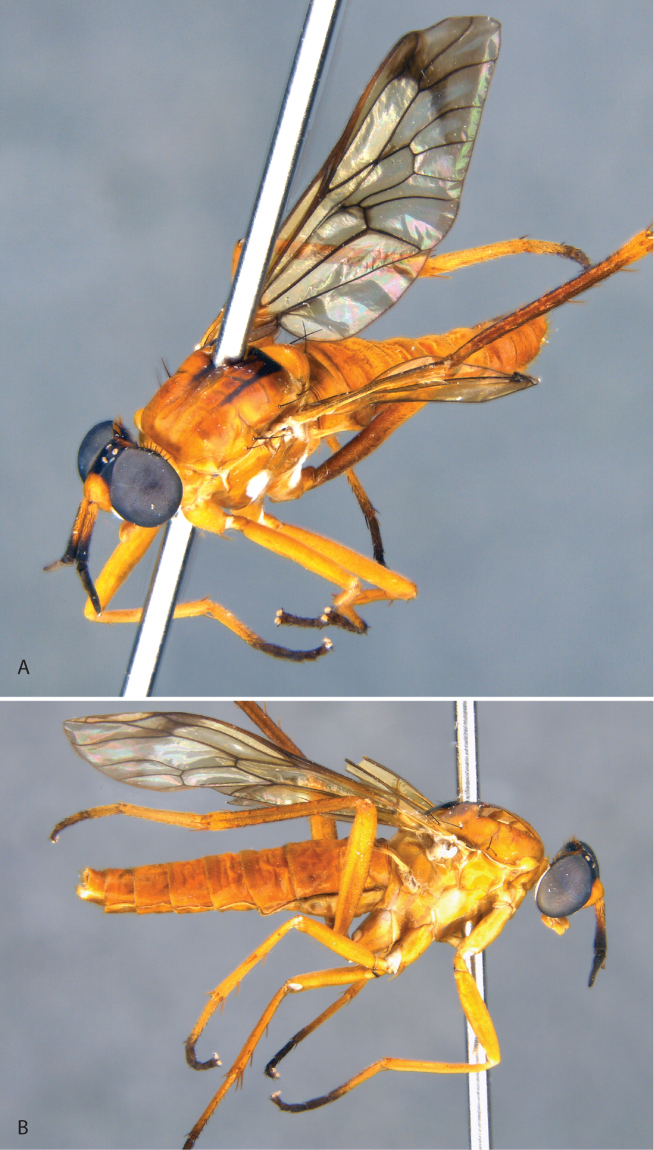
*Calophytusmonteithi* sp. nov. **A** adult female (MEI138464), oblique view **B** same, lateral view. Body length: 10.3 mm.

***Paratypes***. New Caledonia: Province Nord: 1 male, 13 km by road from Ouego[a] to Mont Mandjanié, Malaise trap along stream [-20.374, 164.482], 175 m, 26.XI.1992, D.W. Webb, E.I. & M. Schlinger, (MEI030207, CSCA). Province Sud: 1 male, Réserve Spéciale de Botanique, Mount Ningua, Malaise trap [-21.735, 166.142], 1100 m, 12–21.XII.2000, L.J. Boutin, M.E. Irwin (MEI131365, CSCA); 3 females, Réserve Spéciale de Botanique, Mount Ningua, Malaise trap [-21.735, 166.142], 1100 m, various dates: 9–21.XII.2000, D.W. Webb, E.I. Schlinger, M.E. Irwin (MEI131362–4, CSCA); 2 females, Ningua Reserve Camp, Malaise trap, rainforest [-21.75, 166.15], 1100 m, 12–13.XI.2001, 27.XI–29.I.2002, Burwell, Monteith (MEI138461–2, QM); 2 females, 17 km NNE Nouméa, Mt. Koghis, Malaise trap in tropical forest [-22.167, 166.533], 500 m, 5–15.XI.1992, D.W. Webb (MEI030202–3, CSCA); 2 females, 17 km NNE Nouméa, Mt. Koghis, Malaise trap across forest stream [-22.167, 166.533], 500 m, 23–26.XII.1991, M.E. Irwin, D.W. Webb (MEI030195–6, MEI); 1 female, Nouméa, Mt. Koghis, Malaise trap [-22.167, 166.533], 500 m, 4.XII.1963, R. Straatman (MEI030197, BPBM); 1 female, 17 km NNE Nouméa, Mount Khogis, Malaise trap across path in rainforest [-22.176, 166.505], 425 m, 8.I.1996, M.E. Irwin, D.W. Webb, E.I. Schlinger, (MEI071888, CSCA); 3 females, 30 km NW Yate, Rivière Bleue, Malaise trap [-22.117, 166.658], 12–25.XI.1986, 11–27.X.1988, L.B. de Larbogne, J. Chazeau, A. & S. Tillier, (MEI030198–200, MNHN); 1 female, Rivière Bleue Provincial Park, 19.6 km Rivière Bleue Road, Malaise trap across forest path [-22.117, 166.658], 183 m, 18–20.XI.1992, D.W. Webb (MEI030201, CSCA).

**Figure 16.**
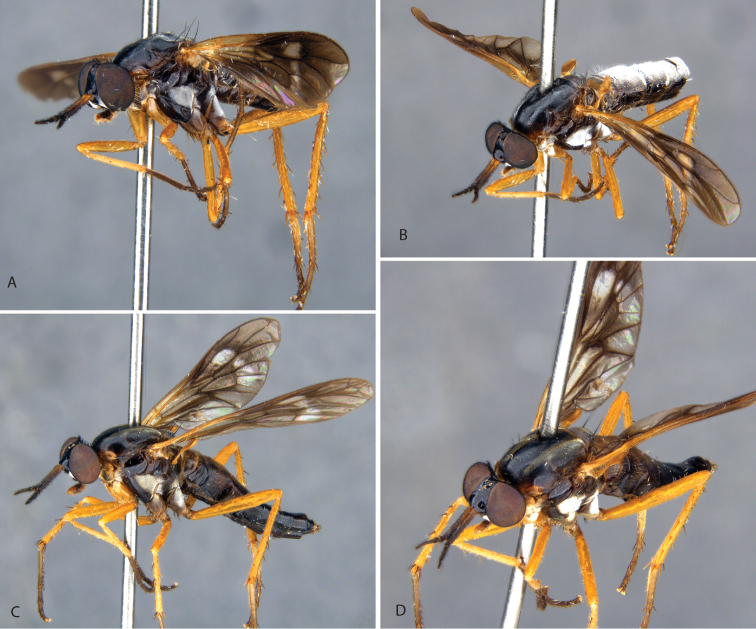
*Calophytusschlingeri* sp. nov. **A** adult male (MEI030158), anterolateral view **B** same, oblique view **C** adult female (MEI030172), lateral view **D** same, oblique view. Body length: male: 8.6 mm; female: 9.8 mm.

**Figure 17.**
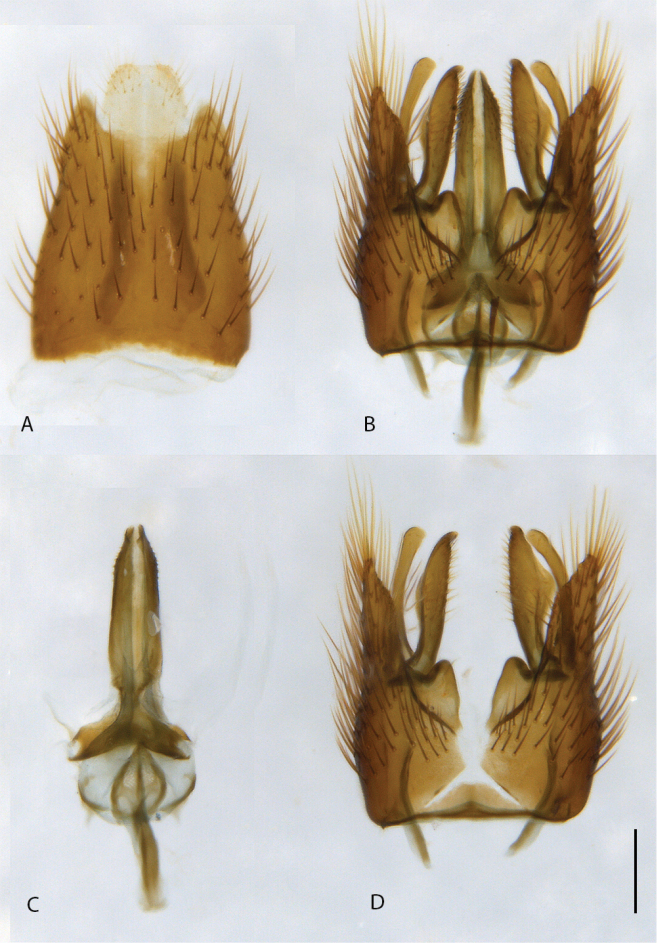
*Calophytusschlingeri* sp. nov., cleared male genitalia **A** epandrium **B** gonocoxites and aedeagus, ventral view **C** aedeagus, dorsal view **D** gonocoxites, ventral view. Scale bar: 0.2 mm.

**Figure 18.**
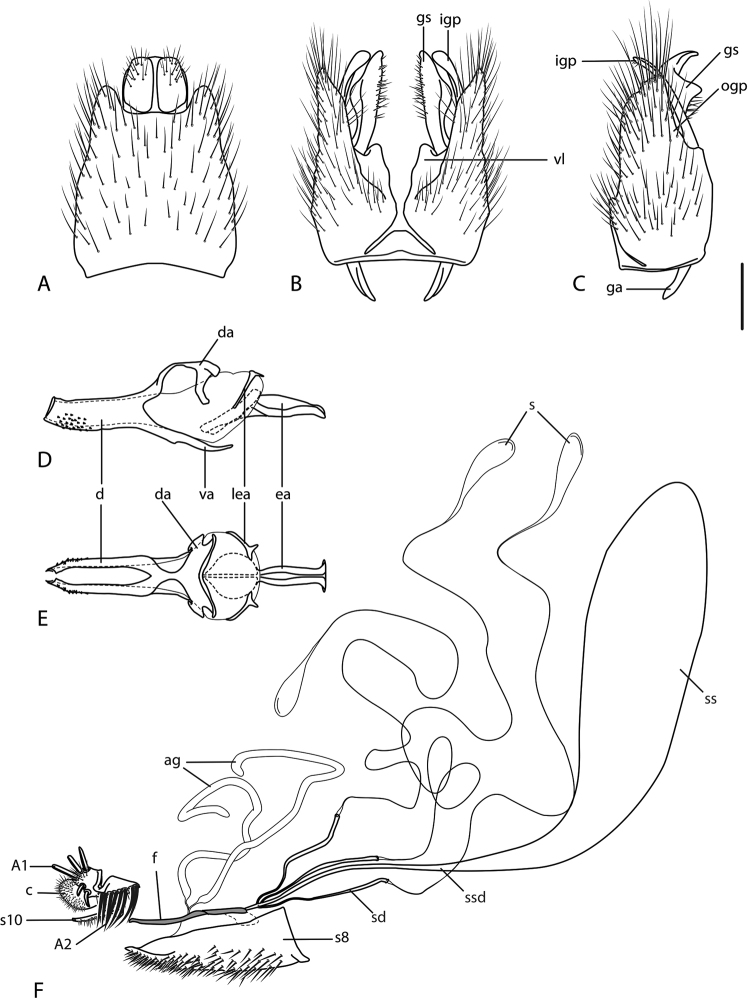
*Calophytusschlingeri* sp. nov., genitalia **A** epandrium **B** gonocoxites, ventral view **C** same, lateral view **D** aedeagus, lateral view **E** same, dorsal view **F** female genitalia, lateral view, internal structures shown. Scale line: 0.2 mm. Abbreviations: *ag*, accessory gland; *d*, distiphallus; *da*, dorsal apodeme of parameral sheath; *ea*, ejaculatory apodeme; *c*, cercus; *A1*, primary acanthophorite spines; *A2* secondary acanthophorite spines; *ga*, gonocoxal apodeme; *gs*, gonostylus; *igp*, inner gonocoxal process (articulated); *lea*, lateral ejaculatory apodeme; *ogp*, outer gonocoxal process; *s8*, sternite VIII; *s10*, sternite X; *s*, spermatheca; *ss*, spermathecal sac; *ssd*, spermathecal sac duct; *sd*, spermathecal duct; *vl*, ventral lobe.

**Figure 19.**
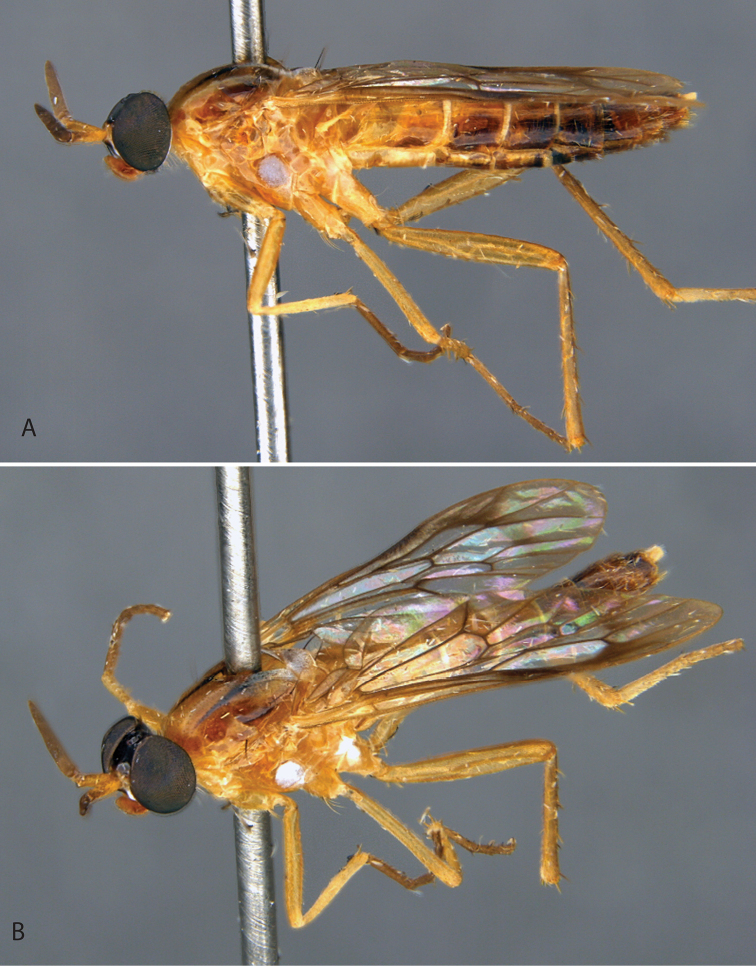
*Calophytuswebbi* sp. nov. **A** adult male (MEI030179), lateral view **B** same, oblique view. Body length: 6.3 mm.

**Figure 20.**
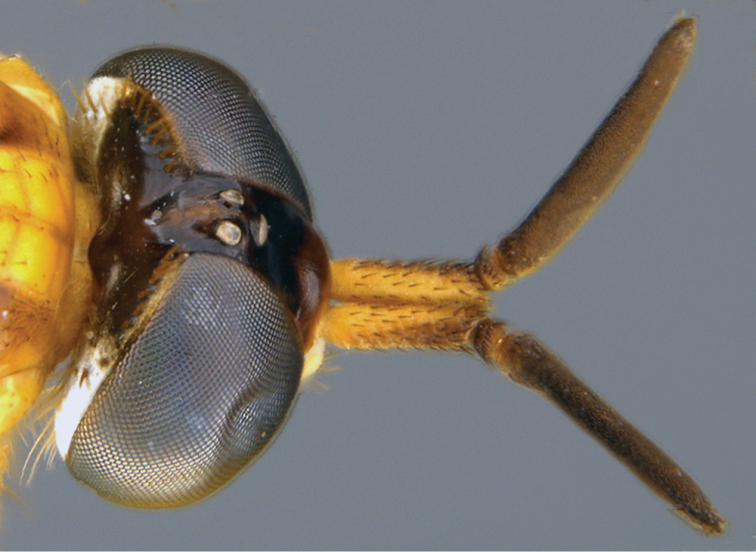
*Calophytuswebbi* sp. nov. adult female head, dorsolateral view.

**Figure 21.**
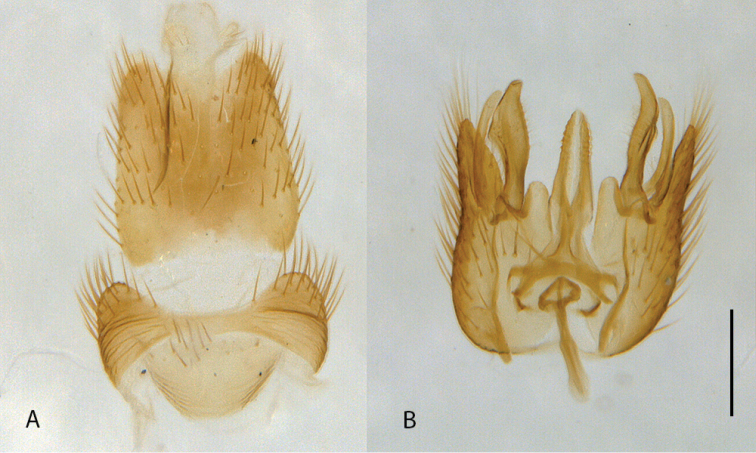
*Calophytuswebbi* sp. nov., cleared male genitalia **A** epandrium and tergite VIII **B** gonocoxites and aedeagus, dorsal view with epandrium removed. Scale bar: 0.2 mm.

**Figure 22.**
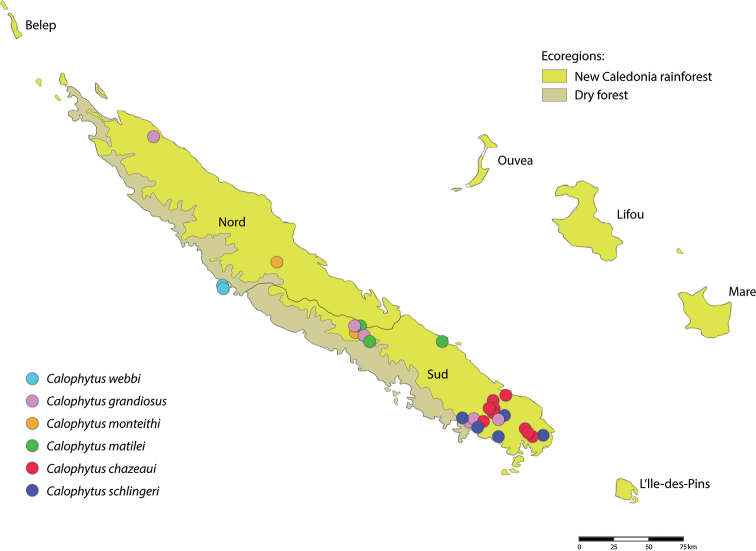
Distribution of *Calophytus* spp. collecting records throughout New Caledonia. Provinces are labelled and ecoregions delineated by colour.

#### Jeanchazeauia rufinatus sp. nov.

Taxon classificationAnimaliaTherevidaeAgapophytinae

D68B4E9D-9324-542F-B315-E9FA75129231

http://zoobank.org/75344FB2-15DD-402C-98CF-45DD58CFEF06

**Figure 23.**
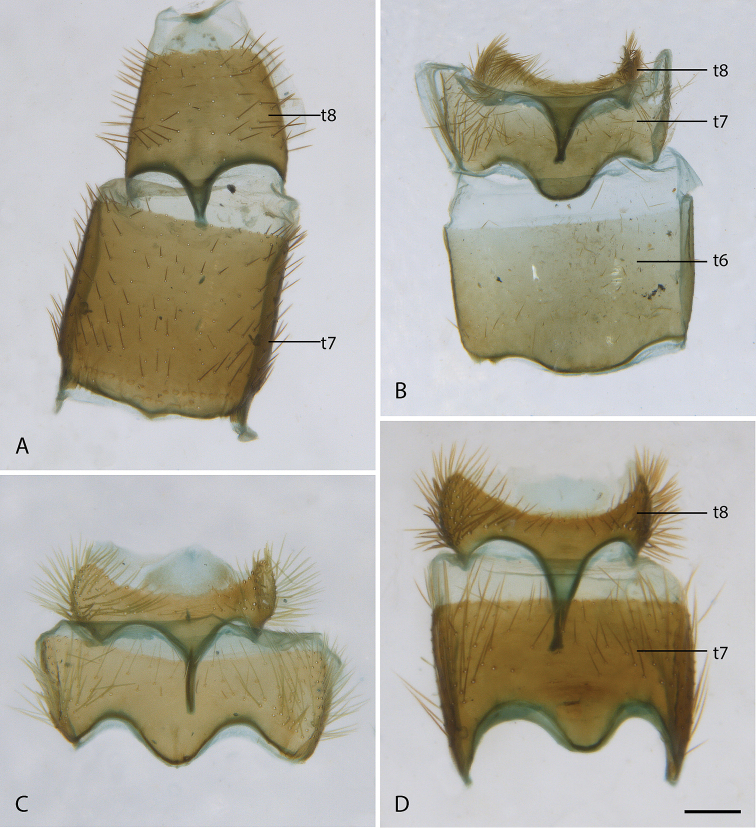
Female abdominal tergites VI–VIII **A***Calophytusschlingeri* sp. nov. **B***Jeanchazeauiaamoa* sp. nov. **C***Jeanchazeauianubilosus* sp. nov. **D***Jeanchazeauiarufinatus* sp. nov. Scale line: 0.2 mm.

Figs 23D , 27G , 28 , 29 , 30

##### Diagnosis.

Female abdomen black with white band on tergite II, tergites IV and V with bright red suffusion; black macrosetae on all femora, posteroventral macrosetae on all femora; frons with silver pubescence; female wing with two regular bands.

##### Description.

Length 10.0 mm. *Head*. Dark brown, overlain with dense silver pubescence. Frons with silver pubescence, dorsal area with faint bronze suffusion at some angles, several short, black, setae below ocellar tubercle, eyes separated by width of ocellar tubercle. Occiput and gena entirely bright silver pubescent, except matte black-bronze pubescence along post ocular ridge; postocular macrosetae mostly black, in a single row dorsally; white setal pile ventrolaterally onto gena. Scape 0.3× head length, dark brown, sparsely pubescent, short, black, setose except medial surface. Basal flagellomere 1.2× length of scape, gradually tapering to a blunt point apically, sparsely silver pubescent with short, black setae dorsally at base. Second flagellomere apical, slightly conical, apex narrower than base; < 1/10× length of basal flagellomere. Third flagellomere ½× length of second, conical. Style small, spiculate. Palpus one segmented, cylindrical, apex slightly capitate; dark brown; silver pubescent, admixed with brown setae. Mouthparts brown; brown setose. *Thorax*. Dark brown, overlain with black and silver pubescence; scutum brown pubescent with broad, silver pubescent dorsocentral stripes converging posteriorly; postpronotal lobes and posterior notopleuron silver pubescent; scutellum black, matte pubescent; pleuron silver pubescent with two brown pubescent vertical bands, first passing from the notum at the prothoracic spiracle posteroventrally through the anterior katepisternum, second band from the wing base posteroventrally through the meron; macrosetae black (np: 2, sa: 1, pa: 1, dc: 0, sc: 1); scutum with short, black setae dorsally; postpronotum, postpronotal lobe, cervical sclerite, proepisternum, and lateral prosternum with at least some short, white setae; katatergite white setose. *Legs*. Dark brown; coxae densely silver pubescent, otherwise sparsely pubescent; short, black setose; all macrosetae black. Fore- and mid coxae admixed with long, black and white setae; hind coxa white setose. Forecoxa with two, midcoxa with four, and hind coxa with five black, anteroventral, marginal macrosetae. Hind femur with one subapical anteroventral macroseta; series of small dark posteroventral macrosetae along middle of all femora, larger on hind femur. *Wing*. Membrane mostly colourless hyaline with two broad dark grey bands; basal band originating at pterostigma and covering membrane directly posterior to posterior wing margin; apical band covering wing tip except apical 1/6 of wing lighter grey; membrane with extensive areas bare of microtrichia. Pterostigma dark grey. Venation dark brown, cell m_3_ wide open at wing margin. Haltere dark brown. *Abdomen*. Dark brown, sparsely silver pubescent, sparsely brown pubescent medially on tergites I–III; tergite II with silver pubescent band on anterior 1/4; tergites IV and V and posterior margin of tergite III reddish orange, except extreme lateral margin dark brown; short, black, setose, apical segments with longer setae; tergites I and II with long, white and black setae laterally. *Genitalia*. Female: tergites VII and VIII highly modified, much wider than long, with an extremely long anteromedial, narrow projection; posterior margin slightly emarginated. Sternite VIII slightly longer than wide, slightly convex ventrally, posterior lobes tapering sharply posteriorly, separate, with a median aedeagal guide; dense, elongate, gold-brown setae present, bare at extreme lateral margin. Medial lobe of tergite IX reduced, very short and only slightly sclerotised, bare of setae. Acanthophorite dark brown; acanthophorite spines dark brown, A2 spines inseparable from rest of acanthophorite setae. Sternite X quadrate, slightly longer than wide, anterior margin truncate, posterior margin slightly emarginate; short, dark brown setose. Furca longer than wide, semicircular anteriorly, tapered to a point posteriorly, origin of spermathecal ducts occurring immediately adjacent to furcal membrane; three spermathecae, much longer than wide, sac-like, wider apically than basally; spermathecal duct thickened basally, extremely narrowed basal to spermathecae; bifurcation of spermathecal sac duct with spermathecal ducts very close to furcal membrane; spermathecal sac duct very long; sac ovoid, longer than wide. Spermathecal ducts and spermathecal sac duct uniting to form a common gonopore on furcal membrane; gonopore wide at orifice and narrowing to common duct.

**Figure 24.**
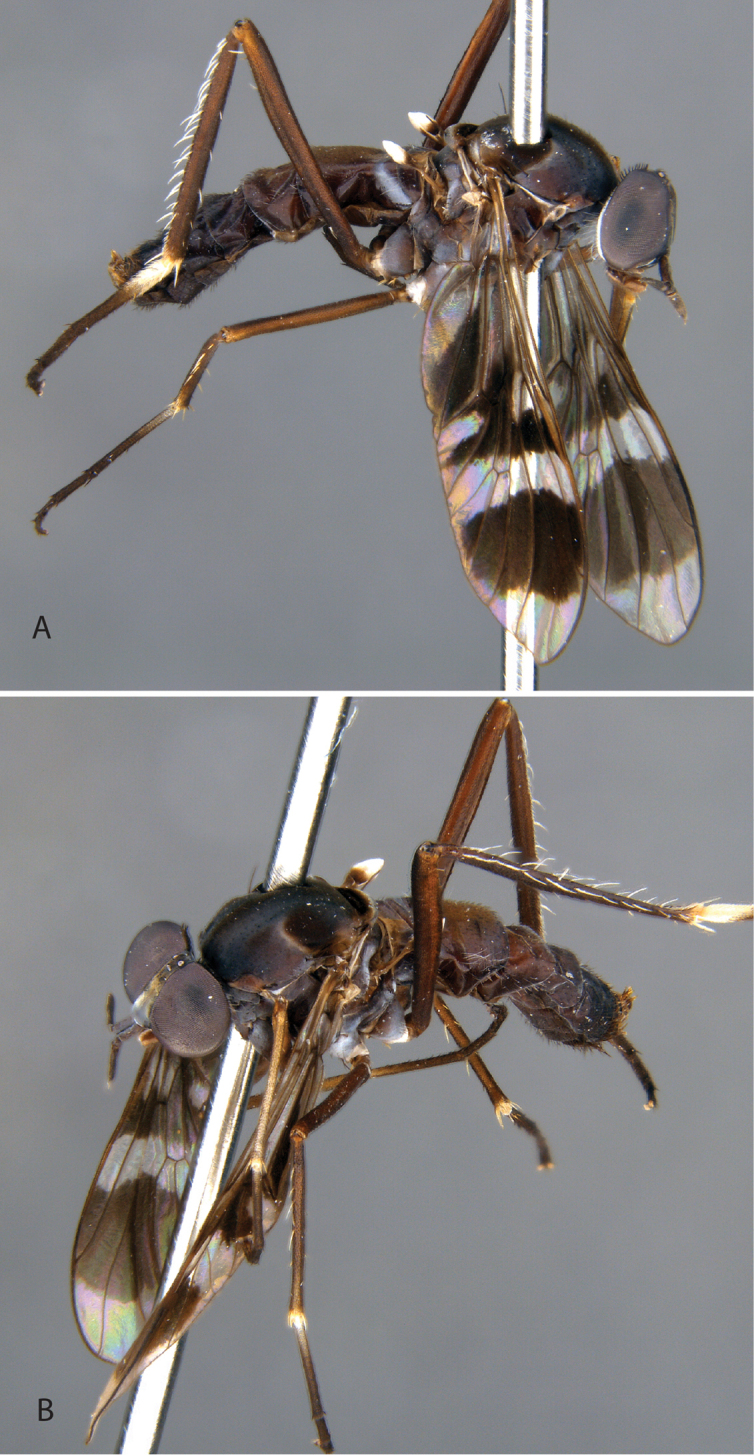
*Jeanchazeauiaamoa* sp. nov. **A** adult female (MEI138463), lateral view **B** same, oblique view. Body length: 7.8 mm.

##### Etymology.

Derived from the Latin *rufus*, red, reddish, and -*atus*, clothed; referring to the reddish area on the female abdomen. Gender is masculine.

**Figure 25.**
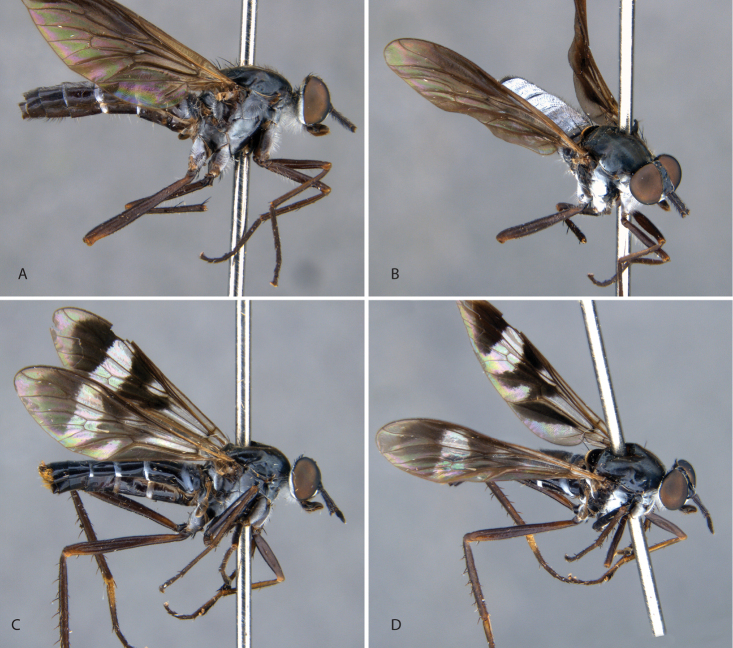
*Jeanchazeauianubilosus* sp. nov. **A** adult male (MEI030207), lateral view **B** same, oblique view **C** adult female (MEI030203), lateral view **D** same, oblique view. Body length: male: 8.8 mm; female: 9.2 mm.

##### Comments.

*Jeanchazeauiarufinatus* sp. nov. can be readily identified by the intensely white pubescence over the entire frons area, and at least in the female by the reddish cuticle on tergites IV and V and on the posterior margin of tergite III. This species has been rarely collected and occurs in rainforest near Sarraméa in the southern province. No males are known.

**Figure 26.**
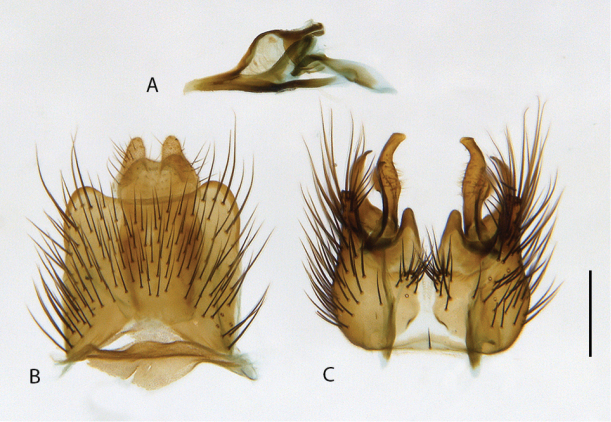
*Jeanchazeauianubilosus* sp. nov. **A** cleared male genitalia **A** aedeagus, lateral view **B** epandrium **C** gonocoxites, ventral view. Scale bar: 0.2 mm.

##### Specimens examined.

***Holotype*** female, New Caledonia: Province Sud: Reserve Col d’ Amieu, 7.5 km NW Sarraméa, Malaise, 21.585°S, 165.819°E [-21.585, 165.819], 4–9.XI.2000, D.W. Webb, E.I. Schlinger, M.E. Irwin, 300 m (MEI108229, MNHN).

**Figure 27.**
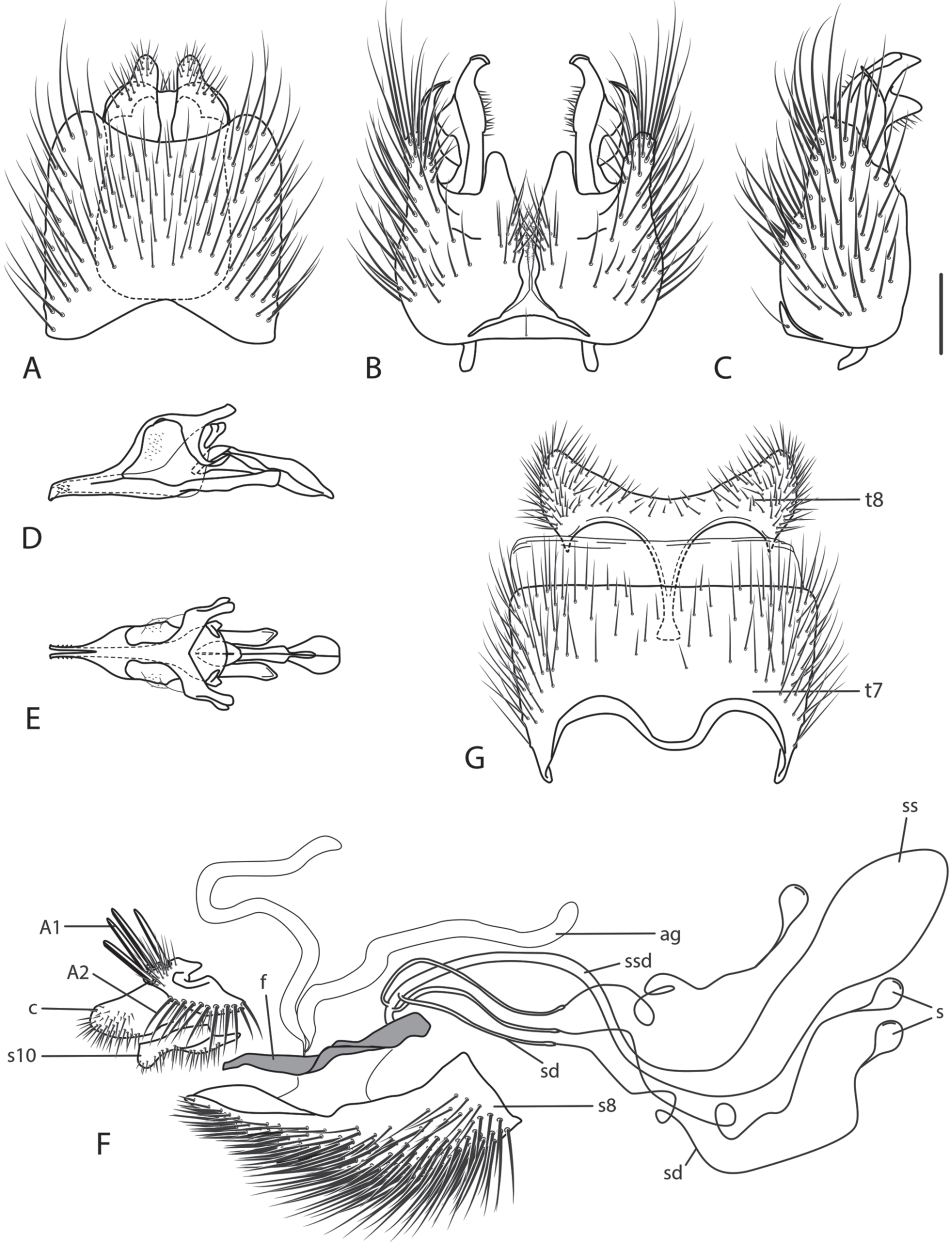
*Jeanchazeauia* spp. genitalia. *Jeanchazeauianubilosus* sp. nov. **A** epandrium **B** gonocoxites, ventral view **C** same, lateral view **D** aedeagus, lateral view **E** same, dorsal view **F** female genitalia, lateral view, internal structures shown. *Jeanchazeauiarufinatus* sp. nov. **G** female tergites VII and VIII, dorsal view. Scale bar: 0.2 mm.

**Figure 28.**
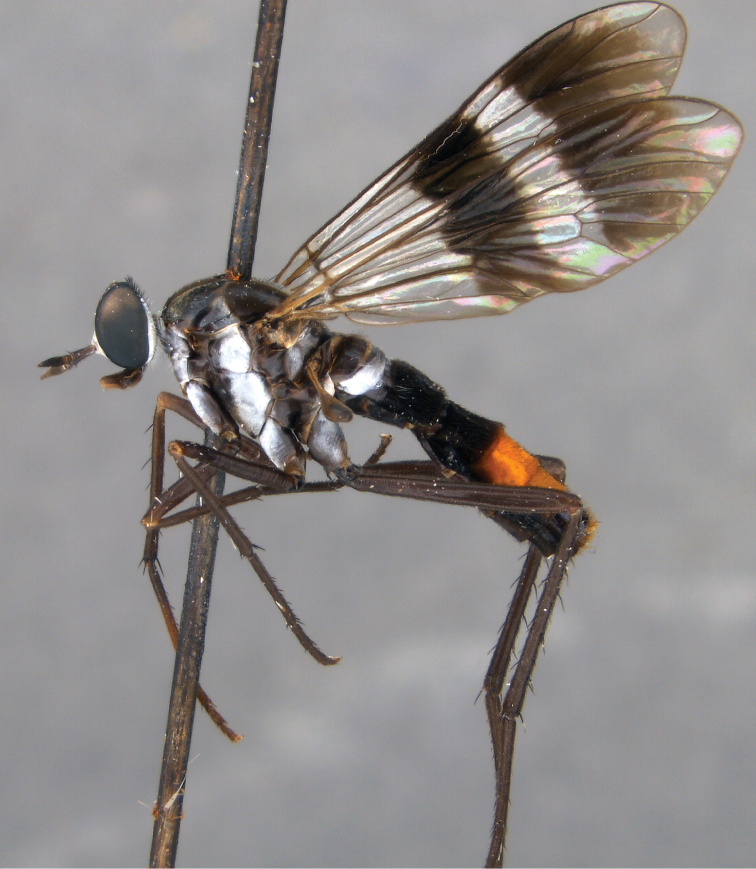
*Jeanchazeauiarufinatus* sp. nov. adult female (MEI071891), lateral view. Body length: 10.0 mm.

**Figure 29.**
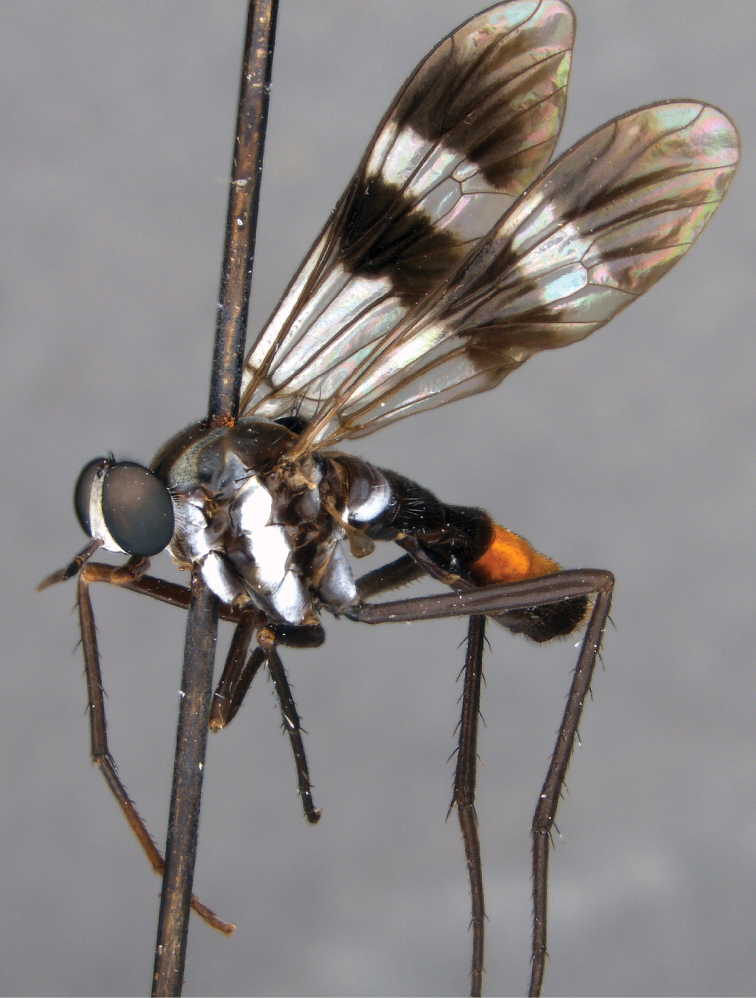
*Jeanchazeauiarufinatus* sp. nov. adult female (MEI071891), oblique view. Body length: 10.0 mm.

***Paratype***. New Caledonia: 1 female Province Sud: 9 km NW Sarraméa, Malaise on forest hillside, 305 m, 9.I.1996, M.E. Irwin, D.W. Webb, E.I. Schlinger, 21°35'07"S, 165°48'55"E [-21.585, 165.815] (MEI071891, CSCA).

**Figure 30.**
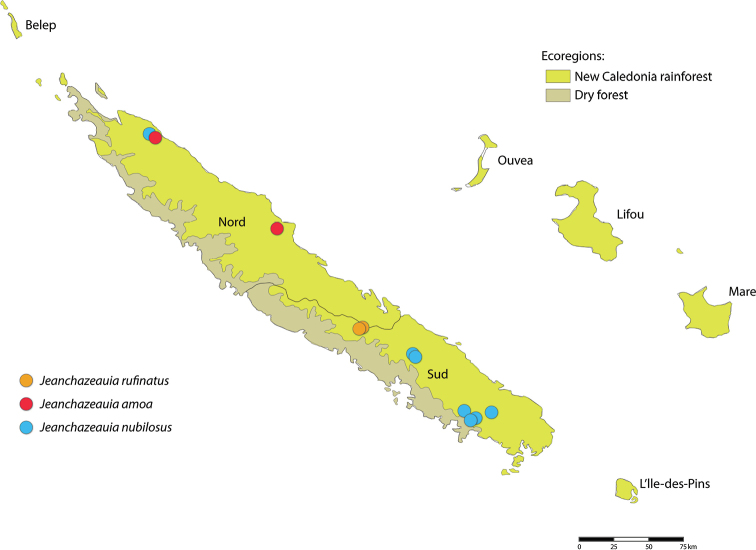
Distribution of *Jeanchazeauia* spp. collecting records throughout New Caledonia. Provinces are labelled and ecoregions delineated by colour.

**Figure 31.**
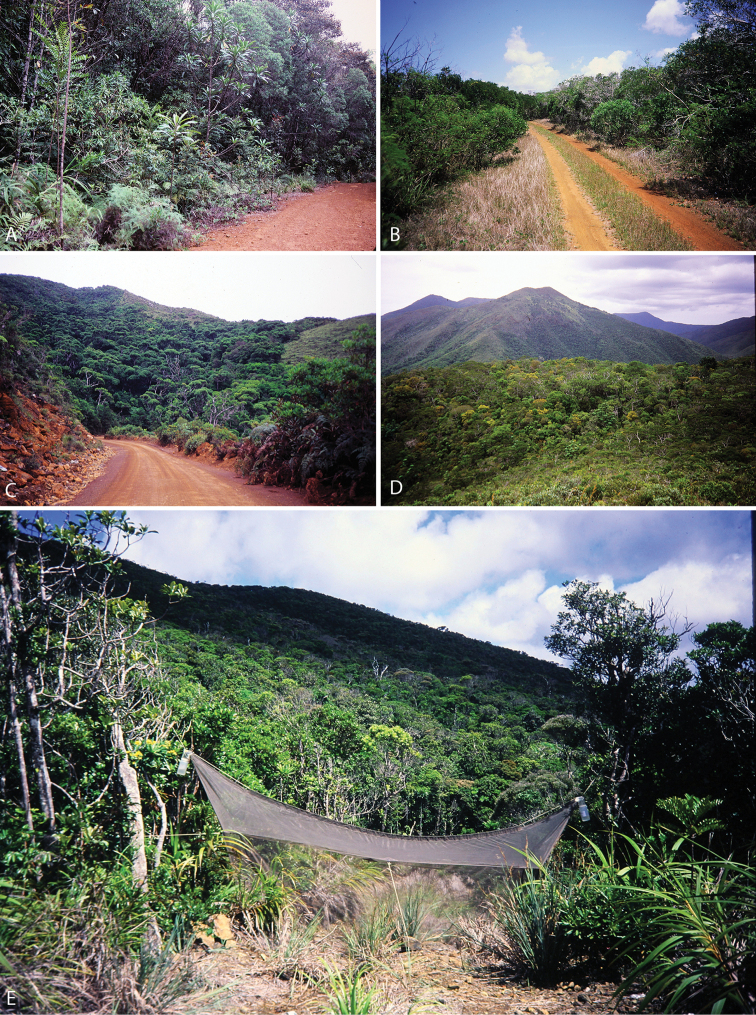
Natural habitats of New Caledonian agapophytine stiletto flies **A** Riviere Bleue Reserve, sclerophyll forest and maquis scrub, habitat of *C.chazeaui* sp. nov., *C.grandiosus* sp. nov., *C.schlingeri* sp. nov., and *J.nubilosus* sp. nov. **B** Pindai Peninsula, tropical dry forest, habitat of *C.webbi* sp. nov. **C** Mount Dzumac, rainforest, habitat of *J.nubilosus* sp. nov. **D** Mount Dore, rainforest and dry sclerophyll forest **E** Mount Dore, Malaise trap in flyway (photographs by Michael E. Irwin).

**Figure 32.**
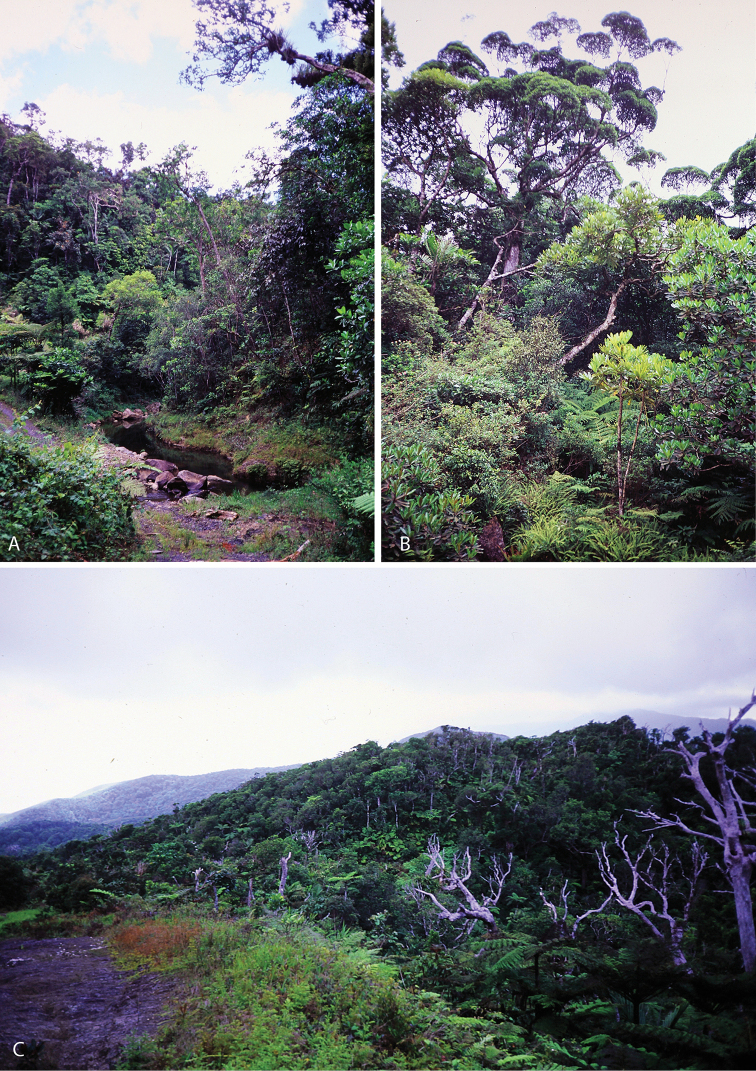
Natural habitats of New Caledonian agapophytine stiletto flies **A** Mount Aoupinie, rainforest, habitat of *C.monteithi* sp. nov. **B** Mount Ningua, rainforest, habitat of *J.nubilosus* sp. nov. **C** Mount Mandjelia, habitat of *C.grandiosus* sp. nov. (photographs by Michael E. Irwin).

## Supplementary Material

XML Treatment for Calophytus

XML Treatment for Calophytus chazeaui

XML Treatment for Calophytus grandiosus

XML Treatment for Calophytus matilei

XML Treatment for Calophytus monteithi

XML Treatment for Calophytus schlingeri

XML Treatment for Calophytus webbi

XML Treatment for Jeanchazeauia

XML Treatment for Jeanchazeauia amoa

XML Treatment for Jeanchazeauia nubilosus

XML Treatment for Jeanchazeauia rufinatus
